# Readiness of the *in vitro* alveolar macrophage assay: an interlaboratory comparison using 12 blinded particles

**DOI:** 10.3389/ftox.2026.1844090

**Published:** 2026-08-28

**Authors:** Emanoela Thá, Antje Vennemann, Vanessa Marani, Oliver Gräb, Andreas Verlohner, Lan Ma-Hock, Inka Marie Spyridonov, Pamina Weber, Aline Chary, Tommaso Serchi, Robert Landsiedel, Martin Wiemann

**Affiliations:** 1 BASF SE, Experimental Toxicology and Ecology, Ludwigshafen am Rhein, Germany; 2 Free University of Berlin, Pharmacy, Pharmacology and Toxicology, Berlin, Germany; 3 IBE R&D Institute for Lung Health gGmbH, Münster, Germany; 4 LIST, Luxembourg Institute of Science and Technology, Esch-sur-Alzette, Luxembourg

**Keywords:** alveolar macrophages, *in vitro* method, inhalation toxicity, NAMs, reproducibility, respirable particles

## Abstract

Inhaled particles can induce persistent pulmonary inflammation when macrophage-mediated clearance mechanisms are overwhelmed. Distinguishing particles that trigger biological activity through intrinsic toxicity from those acting primarily via particle overload represents a central challenge in inhalation toxicology and regulatory safety assessment. Mechanistically anchored new approach methodologies (NAMs) that capture key macrophage responses may therefore provide valuable tools for particle hazard identification while reducing reliance on animal testing. The alveolar macrophage assay (AMA) was developed as an *in vitro* method to assess particle-induced macrophage activation using four biological endpoints reflecting oxidative stress, lysosomal activation, cytotoxicity, and inflammatory signaling. Previous work demonstrated predictivity of the AMA for short-term inhalation outcomes of nanomaterials. However, broader application of the method requires evidence that the assay can be reliably transferred across laboratories and produce reproducible results with a wide variety of particles. In the present study, an interlaboratory ring-study was conducted to evaluate the transferability and reproducibility of the AMA. Twelve blinded particles were tested in three independent laboratories using standardized protocols and predefined acceptance criteria. Across laboratories, comparable dose-response patterns were observed for most endpoints, and seven of the twelve particles were consistently classified as either biologically active or passive according to the established data interpretation procedure (DIP). Sources of variability were identified, including differences in assay sensitivity, and limitations related to dose metrics and partially soluble particles. A main source of variability was the fetal bovine serum (FBS), highlighting the urgent need for transition to animal-component-free and chemically defined culture media to improve reproducibility of *in vitro* methods. Taken together, the results demonstrate that the AMA can be successfully transferred across laboratories and generate reproducible biological responses when strict standardization and quality criteria are applied, which underlines the importance of good *in vitro* method practice (GIVIMP). These findings represent an important step toward the validation of the AMA and support its use as a NAM for mechanism-based hazard assessment and grouping of particles in inhalation toxicology.

## 1 Introduction

Adverse health effects caused by inhaled particles have been recognized for more than a century, particularly in occupational environments with sustained dust exposure (Zenker, 1867; Donaldson and Seaton, 2012; Donaldson et al., 2017). Persistent particles that reach the alveolar region can induce chronic inflammation and tissue damage when pulmonary clearance mechanisms are overwhelmed (Morrow, 1992; Driscoll and Borm, 2020). Particles with an aerodynamic diameter smaller than approximately 4 µm can reach the alveoli (European Committee for Standardization, 1993), where alveolar macrophages (AMs) represent the central defense system responsible for particle uptake, clearance, and the initiation of inflammatory responses (Oberdörster et al., 1992; Liu et al., 2025; Shan et al., 2026).

The macrophage response forms the mechanistic core of particle-induced lung toxicity. When macrophages efficiently clear deposited particles, inflammation remains transient and tissue homeostasis is preserved. However, when particle burdens exceed macrophage clearance capacity, clearance slows and persistent inflammation may develop. This phenomenon is conceptualized in the lung overload paradigm, which explains adverse responses to poorly soluble low-toxicity (PSLT) particles primarily as a consequence of excessive particle retention rather than intrinsic particle toxicity (Morrow, 1988; Borm et al., 2015; Driscoll and Borm, 2020; Stratmann et al., 2021; Ma-Hock et al., 2026). Within this framework, a fundamental distinction emerges between particles that cause biological effects through high retained particle burdens and those that trigger toxicity through specific physicochemical properties. Discriminating between these two mechanisms is a central challenge in inhalation toxicology and regulatory assessments.

The rapid emergence of engineered nanomaterials has intensified this challenge. Nanomaterials exhibit a wide diversity of physicochemical properties and large surface-area-to-mass ratios that can alter their biological interactions relative to larger particles (Oberdörster et al., 2005; Gottardo et al., 2017; Riediker et al., 2019). Consequently, many particulate materials now require toxicological assessment under regulatory frameworks such as the European Union’s REACH regulation. At the same time, regulatory policy increasingly emphasizes that animal studies should be used only when necessary, consistent with the principles of replacement, reduction, and refinement (3Rs) (European Commission, 2025). These developments create a strong demand for human-relevant testing strategies capable of evaluating particle toxicity while minimizing animal testing.

New approach methodologies (NAMs) are therefore becoming an increasingly important component of modern toxicology (Clippinger et al., 2018; Stucki et al., 2022; Haber et al., 2024). For particulate materials, NAMs should ideally capture the key biological processes underlying particle-induced lung toxicity. In particular, macrophage activation, oxidative stress responses, and inflammatory mediator release represent central events in the proposed adverse outcome pathways associated with particle exposure (ECETOC, 2013; Bos et al., 2019; Li et al., 2022). Mechanistically anchored *in vitro* assays targeting these processes therefore offer the potential to support early hazard identification and contribute to grouping and prioritization strategies for particulate materials.

One such assay is the alveolar macrophage assay (AMA), which was developed as a mechanistically informed *in vitro* method to evaluate the biological activity of inhaled particles. Building on earlier work by Rehn and colleagues (1999), Wiemann et al. (2016) established the AMA using the rat alveolar macrophage cell line NR8383. The assay measures four complementary endpoints: hydrogen peroxide (H_2_O_2_) formation, β-glucuronidase (GLU) release, lactate dehydrogenase (LDH) release, and tumor necrosis factor-α (TNFα) production. Together, these endpoints reflect key macrophage responses to particle exposure, including oxidative stress, lysosomal activation, cytotoxicity, and inflammatory signaling.

The AMA was designed to distinguish particles with intrinsic toxicity from particles that primarily act through macrophage overload. In the original study, a prediction model based on a “two-out-of-four” decision rule was introduced. According to this framework, particles are classified as biologically active if at least two endpoints show statistically significant responses below a predefined surface area-based dose threshold. It should be mentioned that most of the nanomaterials used in that study formed small agglomerates prone to gravitational settling so that cells had a chance to quantitatively phagocytose the test particles. Using this approach, the AMA successfully predicted the outcomes of standardized short-term inhalation studies (STIS) (Klein et al., 2012; Takebayashi et al., 2019) for a panel of nanomaterials with high sensitivity and specificity (Wiemann et al., 2016).

Beyond its initial development, the AMA has also been incorporated into particle grouping strategies such as DF4nanoGrouping (Arts et al., 2015; Arts et al., 2016; Janer et al., 2021a; Janer et al., 2021b). Within these frameworks, the assay contributes biological information that complements physicochemical characterization and helps differentiate biologically active particles from particles that behave as PSLT materials. Such approaches illustrate how mechanistically anchored NAMs can support regulatory decision-making by providing information relevant for grouping, read-across, and targeted testing strategies (ECETOC, 2013; Borm et al., 2015; DGUV, 2020; Driscoll and Borm, 2020; Stratmann et al., 2021; Driscoll, 2022; DGUV, 2023; Ma-Hock et al., 2026).

However, for any new toxicological method, reproducibility and transferability across laboratories are prerequisites for broader acceptance. Demonstrating that an assay produces consistent results when implemented by independent laboratories is therefore a critical step in the validation of NAMs. Ring trials and interlaboratory comparisons provide the empirical evidence required to evaluate assay reliability and to identify potential sources of variability in experimental procedures or biological systems (Jacobs et al., 2024).

The AMA has previously demonstrated good repeatability within individual laboratories and promising predictive capacity for *in vivo* inhalation outcomes. Nevertheless, broader application of the assay requires evidence that the method can be successfully transferred to independent laboratories and that comparable results can be obtained under different experimental conditions.

The objective of the present study was therefore to evaluate the transferability and interlaboratory reproducibility of the AMA in a blinded ring trial involving three independent European laboratories. Twelve particulate materials were tested using standardized protocols and predefined acceptance criteria. By examining assay performance and consistency of particle classification across laboratories, this study provides critical evidence supporting the further validation and potential regulatory use of the AMA as a NAM for inhalation toxicology.

If macrophage-driven mechanisms represent the central determinant of particle-induced lung toxicity, assays capturing these responses must demonstrate robust reproducibility across laboratories. To this end, the present ring trial evaluates whether the AMA can meet this requirement and thereby support grouping and hazard assessment of particulate materials in regulatory toxicology.

## 2 Materials and methods

### 2.1 Study scope and quality

The present study was designed as an international interlaboratory ring trial to evaluate the transferability and reproducibility of the AMA. It was not a formal regulatory validation study under Good Laboratory Practice (GLP). Only one participating laboratory operated under a full GLP quality system, whereas the involvement of academic laboratories without GLP accreditation reflects current practice in early-stage interlaboratory studies for novel *in vitro* methods. Accordingly, the study was conducted under good scientific practice without formal quality assurance unit (QAU) oversight.

To ensure robustness and comparability of results, several quality elements aligned with OECD Good *In Vitro* Method Practices (GIVIMP) were implemented, including harmonized SOPs, use of common test materials, standardized exposure protocols, predefined acceptance criteria, and centralized data evaluation. Interlaboratory variability, including instrument- and kit-specific differences, was therefore considered at the level of qualitative assay performance and particle binary assignment rather than absolute quantitative equivalence, consistent with the exploratory and comparative nature of the ring trial.

### 2.2 Test particles and suspension preparations

The selection of test particles considered (1) data availability (characterization and/or *in vivo* study), and (2) relevance to MACRAMÉ, which focused on advanced materials. Stock dispersions for all three laboratories were prepared by the lead laboratory (Lead Lab). Sample blinding was conducted by Bio-MS (a partner in MACRAMÉ project). Ten of the 12 materials were tested in the concentration range of 22.5–180 μg/mL, apart from SiO_2_ NM-203 and ZnO NM-111, which were tested in a lower dose range (2.8–22.5 μg/mL) due to known toxicity.

The selected dose range of 22.5–180 μg/mL was not arbitrary: it was originally chosen to cover the *in vivo* mass-based dose metrics reported in the inhalation toxicology literature, particularly the work of Rehn et al. (1999), Bruch et al. (2014), and Pauluhn (2009). The highest concentration of 180 μg/mL corresponds to 120 pg/macrophage, which was considered by Bruch et al. (2014) as the *in vivo* upper value. Accordingly, the AMA adopted a mass-based metric to ensure that the *in vitro* exposures reflect physiologically relevant particulate burdens.

For the dispersion, the test particles were weighed in 20 mL glass vials (20 mL Scint-Burk, WHEA986581; Wheaton Industries Inc.) and water was added to obtain concentrations of 2.5 mg/mL (312.5 μg/mL for SiO_2_ NM-203 and ZnO NM-111). Due to different dispersibilities, the dispersion protocols differed for the materials (see Table 1): ZnO NM-111 was dispersed using a probe sonicator (Branson Sonifier 450D, 6% amplitude) for 16 min according to the final protocol for producing suitable manufactured nanomaterial exposure media within the NANOGENOTOX European project (Jensen et al., 2011). All other materials were dispersed using an ultrasonic probe (Vibra cell, Sonics and Materials, USA) for 5 min or 10 s (see Table 1). The stock dispersions were adjusted with water to concentrations of 720 μg/mL or 90 μg/mL.

**TABLE 1:** Characterization and dispersion of tested materials.

Particle	Primary particle size [nm]	DLS size in F-12K media [nm]	Specific surface area [m^2^/g]	Dispersion method	Stock dispersion concentration [µg/mL]	Test concentrations [µg/mL]
Corundum	1400–7500	n.a.	1.7	US, 10 s	720	22.5–180
BaSO_4_ NM-220	25	1820	41	US, 5 min	720	22.5–180
TiO_2_ NM-101	5–6	485	170–316	US, 10 s	720	22.5–180
TiO_2_ NM-105	15–24	362	47	US, 10 s	720	22.5–180
WC	50	362	40	US, 10 s	720	22.5–180
WC-Co	<200	201	5–8	US, 10 s	720	22.5–180
CeO_2_ NM-212	10–20	561	27.2	US, 10 s	720	22.5–180
Mn_2_O_3_	30	312	150	US, 5 min	720	22.5–180
SiC Nanowires	D: 100 – 600	n.a.	n.a.	US, 10 s	720	22.5–180
	L: 10000 – 50000					
Quartz DQ12	73.5	650	9.3	US, 10 s	720	22.5–180
SiO_2_ NM-203	5–30	201	200	US, 5 min	90	2.8–22.5
ZnO NM-111	82	316	15	NANOGENOTOX (Jensen et al., 2011)	90	2.8–22.5

DLS: dynamic light scattering, D: diameter, L: length, US: ultrasonic probe, s: seconds, min: minutes.

Each participating laboratory prepared the working dispersions of the distributed materials before each biological replicate. First, to achieve the correct concentration of salts, the particles were diluted 1:2 in double concentrated Krebs-Ringer-Phosphate-Glucose (KRPG) buffer (129 mM NaCl, 4.83 mM KCl, 0.54 mM CaCl_2_, 16.5 mM NaH_2_PO_4_, 5.5 mM glucose, pH 7.3–7.4) for H_2_O_2_ assay or serum-free Kaighn′s Modification of Ham’s F-12 medium (F-12K, Sigma-Aldrich, cat. no. N3520) for GLU, LDH and TNFα assays. Then, the dispersions were further diluted 1:2 in KRPG buffer or F-12K medium to achieve the test concentrations.

### 2.3 Alveolar macrophage assay (AMA)

The AMA was performed as described by Wiemann et al. (2016) with some modifications. The method will be described briefly in the following sections.

#### 2.3.1 Cell culture

NR8383 cells (rat alveolar macrophage cell line, ATCC - CRL-2192) were cultivated in F-12K media supplemented with 15% of heat-inactivated (30 min at 56 °C) fetal bovine serum (FBS, PAN Biotech, cat. no. P30-3302) and with addition of 2 mM L-glutamine (PAN Biotech, cat. no. P04-80050), 100 U/100 μg/mL penicillin/streptomycin (PAN Biotech, cat. no. P06-07100), and 2.5 g/L sodium bicarbonate. Cells were maintained in a humidified atmosphere at 37 °C and 5% CO_2_. For the subculture procedure, cells were left at room temperature (RT) for approximately 15 min to allow cells to detach. Cells were subcultured weekly at a ratio of 1:2.

For the assays, 3 × 10^5^ cells/well were seeded onto two 96-well plates previously coated with fibronectin (0.01 mg/mL in PBS, PAN Biotech, cat. no. 2705001S) and in a final concentration of 5% FBS. Cells were incubated from 18 to 24 h for stabilization.

#### 2.3.2 H_2_O_2_ formation

After 18 h of stabilization, cells were exposed either to test particle suspensions (100 µL in KRPG buffer), or to KRPG buffer alone serving as the negative control (NC), or to Zymosan (final concentration 360 μg/mL, Merck, cat. no. Z4250) as the positive control (PC). To verify assay performance and the accuracy of the colorimetric measurement, control wells containing 30 µM H_2_O_2_ were additionally included. Next, 100 µL of reaction mix [0.1 mM Ampliflu Red (Merck, cat. no. 90101), 2 mM NaN_3_, and 2 U/mL horseradish peroxidase (Merck, cat. no. P8250) in KRPG buffer] were added to the wells. Note that in this test all particle concentrations administered with the 100 µL KRPG were doubled to compensate for the dilution caused by the 100 µL reagent mix. The plate was incubated at 37 °C and 5% CO_2_ for 90 min. The absorbance was read at 570 nm and 595 nm (reference wavelength). The optical density (OD) measurements were subtracted from cell-free particle controls (for each test concentration) to correct for test material adsorption and light scattering. The H_2_O_2_ concentration in the supernatant was calculated according to Lambert-Beer’s Law and using the molar extinction coefficient of resorufin (54,000 L × mol^−1^ × cm^−1^).

#### 2.3.3 β-Glucuronidase (GLU), lactate dehydrogenase (LDH) and tumor necrosis factor (TNFα) assays

Following 24 h of stabilization, the cells were either exposed to 200 µL/well of test particles in serum-free F-12K, or to 200 µL of serum-free F-12K alone (NC), or to 1% Triton X-100 (PC for LDH and GLU) or 0.5 μg/mL LPS (PC for TNFα). The cells were incubated for 16 h at 37 °C, 5% CO_2_, in a humid chamber to prevent evaporation. After treatment, the supernatant was collected and centrifuged (200 × *g*, 10 min) to remove particles and cell debris. To measure LDH release, 50 µL of the supernatant were added to 50 µL of LDH reaction mix (LDH Cytotoxic Detection Kit, Merck, cat. no. 11644793001) and incubated in the dark at RT for a maximum of 10 min. The reaction was stopped with 50 µL of 1 N HCl. To measure GLU activity, 50 μL of the supernatant were incubated with 50 μL of reaction mix (13.3 mM p-nitrophenyl-β-D-glucuronide, Merck, Germany), 0.1% Triton X-100 in 0.1 M sodium acetate buffer (pH 4.5) for 90 min at 37 °C. The reaction was stopped with 50 µL of 0.2 N NaOH. The absorbance was read at 492 nm and 620 nm (reference wavelength) for LDH, and at 405 nm for GLU. The OD measurements were corrected for test material adsorption and light scattering using cell-free particle controls for each concentration and normalized to the PC value (cells lysed with 1% Triton X-100) set as 100%. Finally, for the TNFα assay, 50 µL was added to wells containing 10 μL FBS, and the samples were frozen at −80 °C until analysis. Technical duplicates were pooled, and ELISA was performed using different kits in each laboratory following the manufacturer’s instructions (Lead Lab: ELISA MAX™ Deluxe Set Rat TNF-a, BioLegend, Inc., cat. no. 438204; Lab1: Rat TNF-alpha Quantikine™ ELISA, R&D Systems®, Inc., cat. no. SRTA00; Lab2: Rat TNF-alpha ELISA Kit, Invitrogen, cat. no. ERA56RBX5).

### 2.4 Acceptance criteria

A test run was considered valid if all acceptance criteria described in Table 2 were fulfilled. These criteria were originally established by the lead laboratory and were slightly adjusted during the transferability phase of the project to accommodate differences among the three participating laboratories. In practice, the labs used not only different measurement devices (e.g., photometers) but also occasionally different assay kits, which resulted in higher variability compared to the lead/developer laboratory.

**TABLE 2:** Acceptance criteria for considering a run valid in the Alveolar Macrophage Assay.

Criterion	Description
*General criteria*	
i.	Values of the cell-free particle controls are consistently smaller than those of the particle-exposed cells
ii.	All calculated standard deviations are below 20% (intra-assay variation). Larger deviations require microscopic inspection. In case of visible damage values from no more than 2 wells per particle and no more than 1 well per particle concentration may be disregarded. Otherwise, the test needs to be repeated
*Assay-specific criteria*	
*H*_*2*_ *O*_*2*_	​
iii.	Cells show only low basal H_2_O_2_ production. By experience, an OD570 value should be around 0.2 in the H_2_O_2_ Assay
iv.	Cells increase their H_2_O_2_ production upon zymosan at least 3-fold compared to the cell control
v.	H_2_O_2_ 30 µM control should always be between 20 and 50 µM
*LDH*	​
iv.	The value of the cell control must not exceed 30% of the Triton X-100 positive control
vii.	Particle administration should not lead to values exceeding the Triton X-100 positive control value by more than 20%
*GLU*	​
viii.	The value of the cell control must not exceed 6% of the Triton X-100 positive control
ix.	Particle administration should not lead to values exceeding the Triton X-100 positive control value by more than 20%
*TNFα*	​
x.	Supernatant of the non-stimulated cell control contains less than 50 pg TNFα/mL
xi.	LPS stimulation increases the values from cell control at least 5-fold
xii.	Quartz DQ12 leads to a concentration-dependent and significant increase in of the TNFα concentration

### 2.5 Data analysis and prediction model

All analyses were performed in R version 4.5.0 (R Core Team, 2025). TNFα standard curves were fitted using a four-parameter log-logistic model. The best-fitting parameterization was selected by Akaike Information Criterion (Akaike, 1974) from four-parameter candidate sigmoidal functions (LL.4, LN.4, L.4, LL2.4) implemented in the drc package (Ritz et al., 2016) version 3.2.0. The 4 PL model is defined by Equation 1:

$$f\left(x\right)=c+\frac{d-c}{1+\exp\left(b\left(\ln x-\ln e\right)\right)}$$ (1)

where b denotes the Hill slope, c and d the lower and upper asymptotes, respectively, and e the inflection point. TNFα concentrations were interpolated from the fitted standard curve.

For LDH, GLU, H_2_O_2_ and TNFα, model fit was assessed via quantile residual analysis (Dunn and Smyth, 1996) using the DHARMa package (Hartig, 2024) version 0.4.7 accessed through the performance package (Lüdecke et al., 2021) version 0.15.1, which indicated that a gamma distribution was most appropriate. Data were therefore analyzed using a generalized linear model (GLM) with a gamma error distribution and a log link, as defined in Equation 2:

$$\ln\left({µ}_{i}\right)=x_{i}^{⊤}\beta ,∼Gamma\left({µ}_{i},\phi \right)$$ (2)

where μi = E(Yi) is the expected response, β the vector of fixed-effect coefficients, xi the vector of dose groups (as factor) per experiment and ϕ the dispersion parameter. Models were fitted with the glm() function from the stats package. Dunnett’s *post hoc* multiple-comparison test was applied to assess statistical significance of each dose group against the control using the emmeans package (Lenth and Piaskowski, 2025) version 2.0.1 with multiplicity adjustment applying the multivariate-t distribution.

The prediction method was based on Wiemann et al. (2016), with slight modifications. In this method, the mass-based lowest effect concentration (LOEC, defined as the lowest particle concentration with a statistically significant increase (p < 0.05) relative to the NC) in each assay for each particle is converted to surface area-based concentrations using the respective test material’s surface area (m^2^/g) determined by the method of Brunauer Teller and Emmett metrics (BET). The use of surface area metrics is based on the large body of scientific data demonstrating the correlation of surface area with the effects of nanomaterials (Oberdörster, 1996; Sager et al., 2008; Braakhuis et al., 2014; Schmid and Stoeger, 2016; Cosnier et al., 2021). The threshold of 6,000 mm^2^/mL was empirically defined by Wiemann et al. (2016), based on *in vivo* data.

Accordingly, the criteria to assign particles as *active* (*i.e.*, particles with intrinsic toxicity) were:

- Two or more out of the four assays included in AMA were statistically significant below concentrations equivalent to 6,000 mm^2^/mL (particle surface area), and
- If there was a significant increase in LDH and GLU, a monotonic dose-response relationship should be present, because it is the only biologically plausible outcome. For TNFα and H_2_O_2_ assay, other types of dose-response patterns were considered acceptable, since pronounced cytotoxicity may affect both the release of TNFα and formation of H_2_O_2_.

Otherwise, particles were assigned as *passive* (*i.e.*, effects due to test material-unspecific cellular overload).

## 3 Results

### 3.1 Phase I: transferability

In Phase I, the Lead Lab provided the standard operating protocols (SOPs) and on-site training. The transferability of the method was assessed by the ability to fulfill the acceptance criteria and tests with the particle controls (i.e., corundum and quartz DQ12). During this phase, basal cytotoxicity levels (LDH) were higher than the acceptable values. Through extensive troubleshooting, the lot of FBS used for routine culturing was identified as the cause. Among four different batches of FBS tested, only cells cultivated in one of them resulted in values within acceptance criteria. After adopting the same FBS lot as used in Lead Lab, LDH levels fell below 25% in the NC (F-12K), and corundum and DQ12 showed the expected effects of *passive* and *active* particles, respectively, in both Lab1 and Lab2 (data not shown).

### 3.2 Phase II: inter-laboratory reproducibility

After the transferability phase, tests with the blinded particles were performed. The particle uptake was confirmed visually, and micrographs after 16 h of exposure in Lead Lab and Lab1 are presented in the Supplementary Material. Model estimates derived from the generalized linear model (see Section 2.5) and 95% confidence intervals are presented in Supplementary Table S2.

To evaluate if the values obtained in the ring trial were within expected ranges, mean control values were compared with the historical control data (HCD) from the last 5 years (2020–2025) in the Lead Lab (Supplementary Table S1). All mean control values between biological replicates were within the minimum and maximum values of the HCD, except for GLU-NC in Lab2, which had a mean value of 5.13%, slightly above the maximum value of 4.92% in the Lead Lab.

Regarding individual control values (from each biological replicate), Lab2 had 4 out of 9 values above the HCD maximum for GLU (9.32, 8.09, 7.51% and 5.84%), and two LDH values above the HCD (31.22% and 39.86%). Although these values were not within the acceptance criteria, they were retained in the overall assessment to enable a meaningful interlaboratory comparison and were discussed on a case-by-case basis below. For TNFα, Lab2 used a kit with the lowest standard calibration point as 82.3 pg/mL, which is below range produced by NR8383 cells for most tested particles (Wiemann et al., 2016). With most measured OD values falling below the standard calibration range, TNFα data of Lab2 was excluded from further analyses.

In Lab1, five TNF-PC (LPS) values exceeded the maximum absorbance range of the microplate reader (OD > 4). Based on extrapolation from the assay-specific standard curves, these values then exceeded the HCD maximum (1,277 pg/mL). However, the purpose of the TNF-PC is to confirm that the NR8383 cells respond to LPS stimulation. Therefore, exceeding the HCD maximum does not affect the validity of the TNFα measurements in Lab1.

**FIGURE 1:**
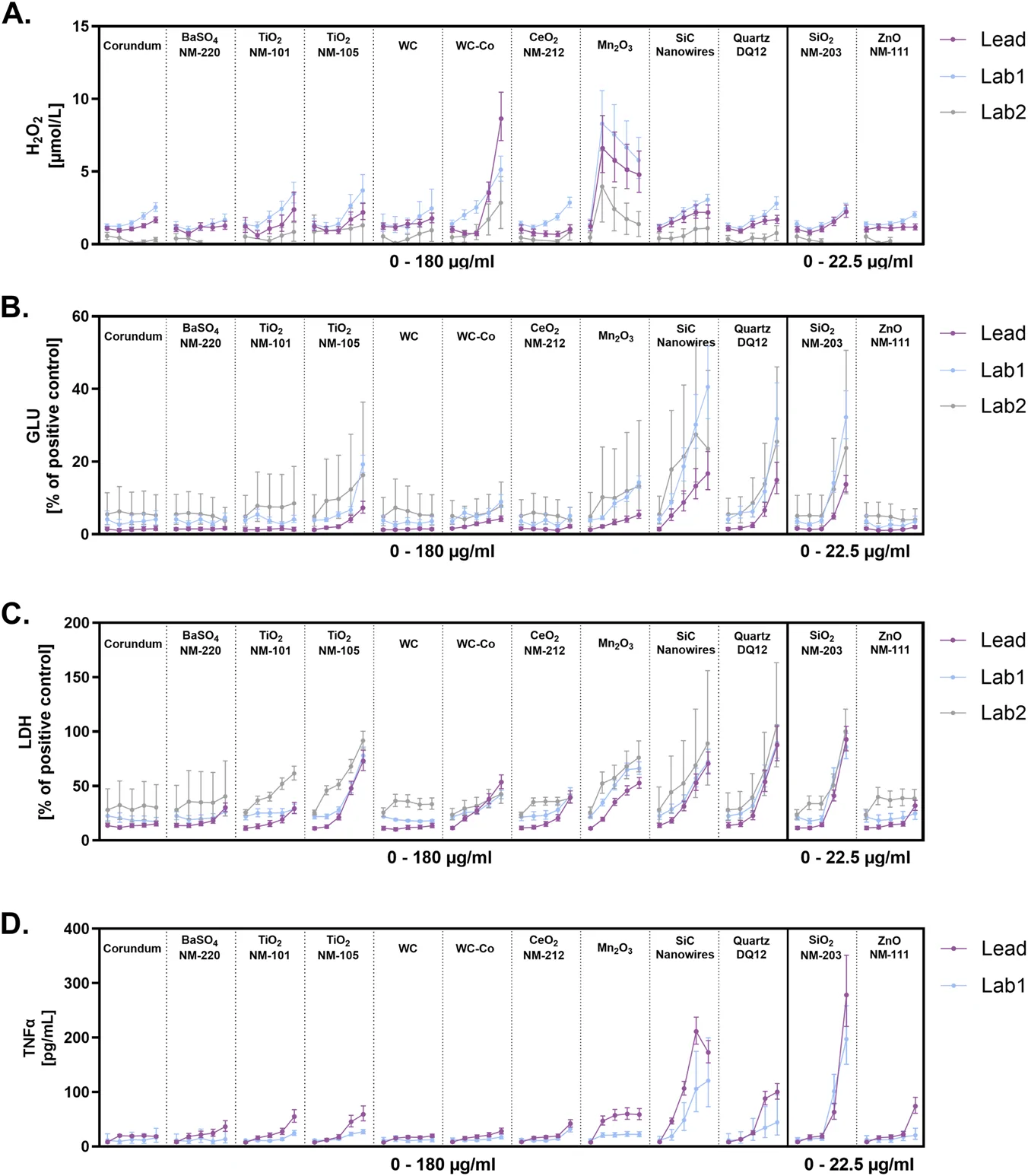
Results obtained in the ring trial across the three participating institutes. Dose responses for all tested particles are shown for the H_2_O_2_ **(A)**, GLU **(B)**, LDH **(C)** and TNFα **(D)** assays. For the TNFα assay, only results from Lead and Lab1 are presented. Each data point represents model estimates ± 95% confidence intervals from three biological repetitions.

When comparing results from all laboratories, similar dose-response patterns were observed for most particles in LDH and GLU assays (Figure 1). For the H_2_O_2_ assay, however, Lab2 obtained negative values after subtraction of the reference wavelength (595 nm), which prevented the analysis for a few concentrations of some particles (Supplementary Table S2).

In Table 3, the LOECs are defined as the lowest particle concentration with a statistically significant increase (p < 0.05) relative to the NC. The LOECs were converted to surface area-based doses using the BET surface area of each particle. Considering the criteria mentioned above and applying the prediction model determined by Wiemann et al. (2016), tested particles were assigned as *passive* or *active*. Due to the absence of reliable TNFα data for Lab2, particles were tentatively assigned as *passive* when no assay was significant below the threshold, and *active* when 2 out of 3 assays showed significant effects below the threshold. Particles with only one positive assay below the threshold were considered as *uncertain*. Seven out of the 12 particles were consistently assigned as either *active* (Mn_2_O_3_, tungsten carbide-cobalt (WC-Co), SiC nanowires, quartz DQ12, and SiO_2_ NM-203) or *passive* (corundum and tungsten carbide (WC)) by all three laboratories (Table 3).

**TABLE 3:** Lowest observed effect concentrations (LOEC) in mass- and surface-area based doses for the three laboratories and the resulting assignment of tested particles.

Particle	BET [m^2^/g]	Lab	LOEC [μg/mL]				LOEC [mm^2^/mL]				Threshold <6,000	Classification
			H_2_O_2_	GLU	LDH	TNFα^#^	H_2_O_2_	GLU	LDH	TNFα^#^		2 out of 4
Corundum	​	Lead	180	n.s.	n.s.	22.5	**306**	n.s.	n.s.	38.25^#^	1	Passive
	1.7	Lab1	90	n.s.	n.s.	n.s.	**153**	n.s.	n.s.	n.s.	1	Passive
	​	Lab2	n.s.	n.s.	n.s.	n.s.	n.s.	n.s.	n.s.	n.s.	0	Passive
BaSO_4_ NM-220	​	Lead	n.s.	n.s.	90	22.5	n.s.	n.s.	**3690**	**922.5**	2	Active
	41	Lab1	n.s.	n.s.	n.s.	n.s.	n.s.	n.s.	n.s.	n.s.	0	Passive
	​	Lab2	n.s.	n.s.	n.s.	n.s.	n.s.	n.s.	n.s.	n.a.	0	Passive
TiO_2_ NM-101	​	Lead	n.s.	n.s.	45	22.5	n.s.	n.s.	10935	**5467.5**	1	Passive
	243	Lab1	90	n.s.	180	180	21870	n.s.	43740	43740	0	Passive
	​	Lab2	n.s.	n.s.	22.5	n.a.	n.s.	n.s.	**5467.5**	n.a.	1	Uncertain
TiO_2_ NM-105	​	Lead	180	22.5	45	22.5	8460	**1057.5**	**2115**	**1057.5**	3	Active
	47	Lab1	90	45	45	90	**4,230**	**2115**	**2115**	**4,230**	4	Active
	​	Lab2	n.s.	n.s.	22.5	n.a.	n.s.	n.s.	**1057.5**	n.a.	1	Uncertain
WC	​	Lead	180	n.s.	n.s.	22.5	7200	n.s.	n.s.	900^#^	1	Passive
	40	Lab1	n.s.	n.s.	n.s.	n.s.	n.s.	n.s.	n.s.	n.s.	0	Passive
	​	Lab2	n.s.	n.s.	22.5	n.a.	n.s.	n.s.	**900** ^*^	n.a.	1	Passive
WC-Co	​	Lead	90	45	22.5	22.5	**585**	**292.5**	**146.25**	**146.25**	4	Active
	6.5	Lab1	22.5	22.5	22.5	n.s.	**146.25**	**146.25**	**146.25**	n.s.	3	Active
	​	Lab2	90	n.s.	90	n.a.	**585**	n.s.	**585**	n.a.	2	Active
CeO_2_ NM-212	​	Lead	n.s.	n.s.	45	22.5	n.s.	n.s.	**1224**	**612**	2	Active
	27.2	Lab1	90	n.s.	90	180	**2448**	n.s.	**2448**	**4,896**	3	Active
	​	Lab2	n.s.	n.s.	22.5	n.a.	n.s.	n.s.	**612** ^*^	n.a.	1	Passive
Mn_2_O_3_	​	Lead	22.5	22.5	22.5	22.5	**3375**	**3375**	**3375**	**3375**	4	Active
	150	Lab1	22.5	45	22.5	22.5	**3375**	6,750	**3375**	**3375**	3	Active
	​	Lab2	22.5	n.s.	22.5	n.a.	**3375**	n.s.	**3375**	n.a.	2	Active
SiC Nanowires	​	Lead	45	22.5	22.5	22.5	n.a.	n.a.	n.a.	n.a.	n.a.	Active
	n.a.	Lab1	22.5	22.5	22.5	45	n.a.	n.a.	n.a.	n.a.	n.a.	Active
	​	Lab2	n.s.	22.5	90	n.a.	n.a.	n.a.	n.a.	n.a.	n.a.	Active
Quartz DQ12	​	Lead	90	45	45	22.5	**720**	**360**	**360**	**180**	4	Active
	8	Lab1	45	90	45	180	**360**	**720**	**360**	**1440**	4	Active
	​	Lab2	n.s.	90	90	n.a.	n.s.	**720**	**720**	n.a.	2	Active
SiO_2_ NM-203	​	Lead	11.25	11.25	5.6	2.8	**2250**	**2250**	**1120**	**560**	4	Active
	200	Lab1	22.5	11.25	11.25	11.25	**4,500**	**2250**	**2250**	**2250**	4	Active
	​	Lab2	n.a.	22.5	2.8	n.a.	n.s.	**4,500**	**560**	n.a.	2	Active
ZnO NM-111	​	Lead	n.s.	n.s.	5.6	2.8	n.s.	n.s.	**84**	**42**	2	Active
	15	Lab1	22.5	n.s.	n.s.	n.s.	**337.5**	n.s.	n.s.	n.s.	1	Passive
	​	Lab2	n.a.	n.s.	2.8	n.s.	n.s.	n.s.	**42** ^*^	n.s.	1	Passive

n.s. = not statistically significantly increased.

n. a. = not assessed.

Bold indicates LOEC below 6,000 mm^2^/mL.

* statistically significant, but not concentration-related, considered not substance-related.

^#^ TNFα, levels in the Lead Lab’s non-exposed controls were consistently low: at least half of those in the lowest particle doses. In this lab, all controls were run on one ELISA plate with the standard curve, while treated samples were measured on separate plates without a standard curve. This likely contributed to the statistically significant increases observed at all doses for all particles, even when no monotonic dose-response was present (e.g., corundum, WC).

Of note, corundum showed an increase in H_2_O_2_ formation at Lead Lab and Lab1. Due to its very low surface area (1.7 m^2^/g), the tested doses do not reach the defined threshold (i.e., 6,000 mm^2^/mL). However, corundum is historically used as a negative benchmark material (Bruch et al., 1993a; Bruch et al., 1993b; Bruch et al., 2014) due to its known insolubility (both in water and biological fluids) and low toxicity, with effects occurring only at high concentrations inducing lung overload (Hartwig and MAK Commission, 2020). For such particles, other metrics other than surface area may be more relevant (see Section 4.4 for detailed discussion). Thus, corundum was maintained as *passive* particle control for AMA, due to its numerous historical control data.

For most materials, Wiemann’s prediction model was applicable; however, this model cannot be used for fiber-like particles such as SiC nanowires. For fibers, number, length and rigidity of fibers are more appropriate dose metrics than surface area (Padmore et al., 2017). Nevertheless, SiC nanowires were provisionally assigned as *active* because they induced concentration-dependent increases in GLU, LDH, and TNFα at concentrations as low as 22.5 μg/mL.

TiO_2_ NM-101, TiO_2_ NM-105, CeO_2_ NM-212, BaSO_2_ NM-220, and ZnO NM-111 showed inconsistent assignments across the three laboratories. TiO_2_ NM-101 was assigned as *passive* and TiO_2_ NM-105 as *active* by both the Lead Lab and Lab 1; however, Lab 2 rated both materials as *uncertain* based solely on a monotonic LDH dose-response without corresponding changes in other endpoints. CeO_2_ NM-212 was assigned as *active* by the Lead Lab and Lab1, but *passive* by Lab2. BaSO_4_ NM-220 and ZnO NM-111 were assigned as *passive* in Lab1 and Lab2, whereas the Lead Lab assigned both as *active*.

## 4 Discussion

The ring trial was conducted to evaluate the transferability and reproducibility of the AMA across three independent laboratories. The study also provided an opportunity to refine SOPs and identify additional factors influencing assay outcomes. With meticulous standardization and strict quality criteria reestablished throughout the study, the reproducibility of the AMA could be demonstrated. The factors affecting the results and the criteria needed for achieving validity of the assay as well as the remaining gaps are thoroughly discussed below.

### 4.1 Main factor influencing assay performance: fetal bovine serum

During the transfer phase, FBS batch was identified as the cause of elevated basal LDH levels in Lab1 and Lab2. Although the assays are performed under serum-free conditions, the routine maintenance of NR8383 cells is dependent on FBS, which is a known source of variability in *in vitro* assays due to undefined composition and batch-to-batch variation (van der Valk et al., 2018; Weber et al., 2025). Along the same line, serum albumin, a primary component of FBS, has been shown to affect cell adhesion (Carré and Lacarrière, 2010; Verdanova et al., 2017). As NR8383 cells are a semi-adherent growing cell line, the FBS could potentially favor one subpopulation in spite of the other. The replacement of FBS by chemically defined animal-free ingredients could improve method reliability and reproducibility. Until such replacement is implemented, testing each FBS batch in the full assay has to be conducted.

### 4.2 Overall reproducibility across endpoints

After the successful transfer, each of the three laboratories tested the 12 blinded particles as suspensions. Overall, the results showed a similar dose-response pattern across the three institutes for all assays, with the exception of TNFα for Lab2. When comparing treatments with p < 0.05, the LDH assay showed the highest concordance across the three institutes. One likely explanation is the use of a well characterized commercial kit, while for GLU and H_2_O_2_ other sources of variability are introduced with the in-house preparation of reagents.

For TNFα, several factors may explain the variability observed between the institutes. While commercially available kits were used, they differed in manufacturer, detection range and sensitivity. Further variability could also arise from plate design and placement of standard curves. Taken together, these factors indicate that, because of the low TNFα production range in NR8383 cells upon particle exposure, a higher standardization would be required to minimize variability and improve reproducibility. This could include specifying assay range and standards, plate layout, and pipetting technique (e.g., forward versus reverse pipetting). These details should be in the future SOP.

### 4.3 Causes for differences across laboratories in the assignment of particles to the groups of passive or active particles

As mentioned before, the negative control values of GLU and LDH of Lab2 exceeded 6% and 30%, respectively, in at least one biological replicate of each particle. We nevertheless decided to retain these data in the analysis to ensure full transparency and to report all values generated within the ring trial. Aware that these elevated controls reduced the dynamic range and introduced additional variability, such values were important contributors to the inconsistent particle assignment. Therefore, the importance of adherence to acceptance criteria should be emphasized. Each mismatch case is discussed below.

TiO_2_ NM-101 and TiO_2_ NM-105 were assigned as *uncertain* in Lab2, whereas the lead laboratory and Lab1 produced consistent assignments (NM-101 as *passive*; NM-105 as *active*). For NM-101, only LDH showed a monotonic, concentration-dependent increase in Lab2; without TNFα, the required second confirming endpoint was missing. For NM-105, Lab2 observed increases in GLU and LDH, but GLU failed to reach statistical significance due to high variability between replicates and inability to meet acceptance criteria for the NC. Similarly, CeO_2_ NM-212 was assigned *active* by the Lead Lab and Lab1, but *passive* by Lab2, since LDH values across concentrations were too similar to meet the monotonic dose-response criterion. Altogether, these discrepancies show that strict standardization of measurement ranges, assay setup, and having runs within the acceptance criteria are essential to ensure consistent assignment across laboratories.

Moreover, ZnO NM-111 also produced divergent results: it was *active* in the Lead Lab based on clear LDH and TNFα increases, but passive in Lab1 and uncertain in Lab2. Likewise, BaSO_4_ NM-220 was only assigned as *active* in the Lead Lab, where slight but concentration-dependent increases in TNFα and LDH were observed. Although this meets the “two out of four” criteria, the magnitude of the responses was minimal and of questionable biological relevance. In contrast, both Lab1 and Lab2 assigned BaSO_4_ as *passive*.

A potential explanation for the mismatch in the assignment of BaSO_4_ NM-220 and ZnO NM-111 might be related to the dynamic dissolution of these particles. BaSO_4_ NM-220 and ZnO NM-111, although poorly soluble in cell-free pH neutral environments, are cleared from the lungs much faster than other poorly soluble particles (Molina et al., 2019; Thoma et al., 2024). In phagolysosomal simulant fluid (PSF, pH 4.5), the calculated clearance half time for BaSO_4_ NM-220 was only about 7 days and that of ZnO NM-111 was 1.2 days in dynamic dissolution assay. For comparison, the clearance half time of CeO_2_ NM-212 and TiO_2_ NM-105 were > 146 and >4,356 days, respectively (Koltermann-Jülly et al., 2018). Thus, for BaSO_4_ NM-220 and ZnO NM-111, toxicity may therefore be driven by the released ions.

There were also further possibilities that may cause this mismatch. For example, the low reactivity of BaSO_4_ makes it a borderline case, consistent with earlier *in vivo* inhalation studies in rats (Konduru et al., 2014; Landsiedel et al., 2014). This indicates a potential disadvantage in the binary classification system and supports the implementation of a borderline or ambiguous category to account for particles inducing minimal but statistically significant responses, as well as particles with responses near the defined threshold. In addition, if BaSO_4_ is kept long in pH-neutral media, it may undergo transformation such as Ostwald ripening, a process in which smaller particles dissolve and recrystallize onto larger particles, leading to an increase in particle size, a reduction in total surface area, and altered toxicological behavior (Keller et al., 2020). Since the Lead Lab performed the test earlier while the other laboratories required more time for transferring and implementing the method, this delay may have affected the particle size in suspensions, potentially altering their properties.

Moreover, ZnO NM-111 had to be tested at lower concentrations based on previous data obtained at the lead laboratory. However, the tested concentration range may have been insufficient to detect effects below the 6,000 mm^2^/mL threshold in Lab1 and Lab2. In these less experienced laboratories, high basal levels and variability of LDH and TNFα measurements limited assay sensitivity. Consequently, effects became apparent only in the lead laboratory, which generated highly reproducible data with low standard deviation.

### 4.4 Remaining gaps

FBS was identified as an important source of variability during the ring trial. Beyond the scientific concerns related to this variability, the use of FBS also raises ethical issues (Rosolowski et al., 2025; Weber et al., 2025). As such, full replacement of animal use will only be achieved when *in vitro* methods are entirely based on defined, animal-component-free media. Therefore, replacing FBS should be pursued in the future to make the AMA a truly animal-free method.

The AMA is performed using a rat alveolar macrophage cell line, NR8383, which is convenient due to its wide availability and unlimited lifespan. In addition, it allowed direct comparison of its results to those of rat experiments. However, human AMs are about four times larger by volume than rat macrophages (about 5,000 and 1,200 μm^3^, respectively (Krombach et al., 1997)). Furthermore, rats are known to be more susceptible than other species, including humans, to particle-induced lung overload and its downstream consequences (Driscoll and Borm, 2020). Therefore, using these cells may overestimate human sensitivity (Scott et al., 2025).

To improve human relevance, the assay needs to be transferred to human macrophages. At present, this remains challenging since there is no human alveolar macrophage cell line available. Human primary AMs can be difficult to obtain and are terminally differentiated and non-proliferative (David et al., 2025). This is a challenge for AMA, as it requires a large number of cells, which may not be broadly available with reproducible quality. One opportunity lies in induced pluripotent stem cells (iPSCs), which could be expanded and differentiated into AMs for the assay. However, the differentiation of these cells to the AM phenotype at scalable and reproducible yields is still under development (Schinke et al., 2025). Thus, the search for the ideal human cell for application in this assay is ongoing (Kelsall et al., 2025; Scott et al., 2025).

The relation of *in vitro* particle concentrations to *in vivo* lung burdens (which in turn are related to the inhaled aerosol concentrations) and its relevance to the *in vitro* assays has been described (Ma-Hock et al., 2021; Azizah et al., 2024; Landsiedel et al., 2024). Yet some open questions remain: the most appropriate dose metric for the assay remains to be determined. There has been debate on which metric is the most relevant for inhalation toxicity of particles (Schmid et al., 2009; Pauluhn, 2011). Surface area appears to be the most biologically relevant dose metric for NMs with surface reactivity, since they have extremely high specific surface area per unit mass, and their effects are often driven not by the absolute particle mass but by the total surface area. On the other hand, volume correlates better with effects caused by chronic exposure to high particle doses (overload effects) (Pauluhn, 2014; Schmid and Stoeger, 2016), such as for corundum.

Rehn et al. (1999) set their *in vitro* particle doses to reflect the mass range of particulate burdens that AMs are likely to encounter *in vivo* in rats. Similarly, Wiemann et al. (2016) applied the same concept with the addition of retrospective conversion to surface area doses, based on the large body of work demonstrating the correlation of this dose metric with NM effects (Oberdörster, 1996; Sager et al., 2008; Braakhuis et al., 2014; Schmid and Stoeger, 2016; Cosnier et al., 2021). To maintain methodological continuity with the original assay development and to avoid the introduction of uncertainties in assay performance, the authors deliberately retained the previously published four mass-based dose levels.

The volume-based metric is consistent with classical overload concepts, such as Morrow’s proposed threshold of ∼60 μm^3^ particle volume per rat AM, which describes the point at which macrophage activity begins to be impaired (Morrow, 1988; Oberdörster, 1988). Accordingly, volume-based dose metrics could provide a more mechanistically relevant basis for predicting lung overload and distinguishing intrinsic toxicity from effects driven purely by particle burden. Characterization data on the particle agglomerate density allowed retrospective conversion to volume-equivalent metrics (Supplementary Table S3). However, for the majority of particles examined, even the highest concentration used in the AMA (180 μg/mL) did not correspond to the particle volume of ∼60 μm^3^ per cell. As a result, the study design did not reach particle burdens high enough to meaningfully test volumetric overload conditions. Consequently, although volume-based dosimetry provides valuable mechanistic insight, the applicability of overload theory could not be evaluated within the current experimental confines and should be specifically addressed in future studies through adjusted dosing strategies or extended dose ranges. With this, the prediction model could be further refined to also account for particles with low surface area, such as corundum.

### 4.5 Readiness level

Technology Readiness Levels (TRLs) were originally developed by NASA to describe the maturity of a technology from early concept to routine operational use (NASA, 2017). For toxicological methods and NAMs, the relevant “technology” is not the cell model alone, but the complete method: biological test system, exposure procedure, SOPs, controls, acceptance criteria, analytical endpoints, data analysis, prediction model, applicability domain and context of use (Worth and Balls, 2001; European Commission, 2014; Cöllen et al., 2024).

The AMA has progressed beyond typical TRL 6–7 characteristics. It is no longer an exploratory assay or a prototype demonstrated only under developer-laboratory conditions. The method has a defined mechanistic rationale, standardized procedures, predefined acceptance criteria, benchmark materials, a structured data interpretation procedure and prior evidence of relevance for short-term inhalation outcomes (Wiemann et al., 2016). The present interlaboratory comparison adds the critical next step: it shows that the assay can be transferred to independent laboratories and can generate reproducible biological response patterns when strict standardization and quality criteria are applied, consistent with GIVIMP principles (Bal-Price et al., 2018; OECD, 2018). Importantly, this interlaboratory demonstration extends the method beyond proof-of-concept in a single laboratory and supports operation under relevant conditions across independent sites. The available evidence therefore supports positioning the AMA within the TRL 7–8 range within its defined context of use. This context is the assessment and grouping of respirable particulate materials based on macrophage-mediated biological activity. Within this domain, the assay represents a qualified NAM candidate rather than a research model. It provides a standardized, mechanistically anchored and decision-oriented method that can contribute to integrated particle safety assessment and grouping frameworks, including DF4nanoGrouping and the ECETOC NanoApp (Arts et al., 2015; Arts et al., 2016; Janer et al., 2021a; Janer et al., 2021b).

The present study also indicates that the prediction model could be revised, particularly with respect to dose metrics, partially soluble materials, low-surface-area particles, borderline responses and fiber-like materials. This does not question the reproducibility or technical transferability of the assay. It concerns the interpretation of how biological responses are converted into particle classifications across different material classes. Refining the prediction model is therefore part of the maturation of the AMA, not evidence of a technical deficiency.

The AMA has not yet reached TRL 9. It has not been adopted as an OECD Test Guideline and has not yet been established as a routinely accepted regulatory method across its full intended operational scope. Taken together, the current evidence places the assay within the TRL 7–8 transition range, reflecting demonstrated transferability and partial reproducibility across laboratories, while indicating that further refinement is required to meet all criteria associated with TRL 8. Further work should focus on revising the prediction model, exploring a wider applicability domain, harmonizing remaining assay components and building regulatory confidence.

## 5 Conclusion

Overall, this ring trial demonstrates that AMA yields reproducible results when strict standardization and adherence to quality measures are ensured. Several factors, including FBS batch variability and TNFα assay sensitivity, were identified as causes for variability and require further optimization. Gaps that remained to be filled also include the identification of appropriate dose metrics and the transition to a more human relevant and truly animal-free assay through the use of human cells and animal-component-free media. Therefore, our work paves the way for future research focusing on establishing serum-free or chemically defined culture media, fully harmonizing TNFα measurements, integrating surface area or volume metrics into dose selection and transferring the assay to human cells. By establishing robust interlaboratory transferability and reproducibility, this study provides key evidence needed for the formal validation and regulatory uptake of the AMA as a NAM. It positions the AMA for integration into inhalation safety assessment frameworks for regulatory decision-making on particle-induced lung toxicity.

## Data Availability

The original contributions presented in the study are included in the article/Supplementary Material, further inquiries can be directed to the corresponding author.

## References

[B1] Akaike H. (1974). A new look at the statistical model identification. IEEE Trans. Automatic Control 19 (6), 716–723. 10.1109/TAC.1974.1100705

[B2] Arts J. H. Hadi M. Irfan M. A. Keene A. M. Kreiling R. Lyon D. (2015). A decision-making framework for the grouping and testing of nanomaterials (DF4nanoGrouping). Regul. Toxicol. Pharmacol. 71 (2 Suppl. l), S1–S27. 10.1016/j.yrtph.2015.03.007 25818068

[B3] Arts J. H. Irfan M. A. Keene A. M. Kreiling R. Lyon D. Maier M. (2016). Case studies putting the decision-making framework for the grouping and testing of nanomaterials (DF4nanoGrouping) into practice. Regul. Toxicol. Pharmacol. 76, 234–261. 10.1016/j.yrtph.2015.11.020 26687418

[B4] Azizah R. N. Verheyen G. R. Shkedy Z. Van Miert S. (2024). Overview of in vitro-in vivo extrapolation approaches for the risk assessment of nanomaterial toxicity. NanoImpact 35, 100524. 10.1016/j.impact.2024.100524 39059748

[B5] Bal-Price A. Hogberg H. T. Crofton K. M. Daneshian M. FitzGerald R. E. Fritsche E. (2018). Recommendation on test readiness criteria for new approach methods in toxicology: exemplified for developmental neurotoxicity. ALTEX 35 (3), 306–352. 10.14573/altex.1712081 29485663 PMC6545888

[B6] Borm P. Cassee F. R. Oberdörster G. (2015). Lung particle overload: old school -new insights? Part Fibre Toxicol. 12, 10. 10.1186/s12989-015-0086-4 25927223 PMC4419487

[B7] Bos P. M. J. Gosens I. Geraets L. Delmaar C. Cassee F. R. (2019). Pulmonary toxicity in rats following inhalation exposure to poorly soluble particles: the issue of impaired clearance and the relevance for human health hazard and risk assessment. Regul. Toxicol. Pharmacol. 109, 104498. 10.1016/j.yrtph.2019.104498 31604110

[B8] Braakhuis H. M. Park M. V. Gosens I. De Jong W. H. Cassee F. R. (2014). Physicochemical characteristics of nanomaterials that affect pulmonary inflammation. Part Fibre Toxicol. 11, 18. 10.1186/1743-8977-11-18 24725891 PMC3996135

[B9] Bruch J. Rehn B. Song H. Gono E. Malkusch W. (1993a). Toxicological investigations on silicon carbide. 1. Inhalation studies. Br. J. Ind. Med. 50 (9), 797–806. 10.1136/oem.50.9.797 8398873 PMC1061312

[B10] Bruch J. Rehn B. Song W. Gono E. Malkusch W. (1993b). Toxicological investigations on silicon carbide. 2. *in vitro* cell tests and long term injection tests. Br. J. Ind. Med. 50 (9), 807–813. 10.1136/oem.50.9.807 8398874 PMC1061313

[B11] Bruch J. Rehn B. Duval-Arnould G. Efskind J. Roderer G. Sebastian P. (2014). Toxicological investigations on the respirable fraction of silicon carbide grain products by the *in vitro* vector model. Inhal. Toxicol. 26 (5), 278–288. 10.3109/08958378.2014.885099 24669950

[B12] Carré A. Lacarrière V. (2010). How substrate properties control cell adhesion. A physical–chemical approach. J. Adhesion Sci. Technol. 24 (5), 815–830. 10.1163/016942409X12598231567862

[B13] Clippinger A. J. Allen D. Jarabek A. M. Corvaro M. Gaca M. Gehen S. (2018). Alternative approaches for acute inhalation toxicity testing to address global regulatory and non-regulatory data requirements: an international workshop report. Toxicol Vitro 48, 53–70. 10.1016/j.tiv.2017.12.011 29277654 PMC8215693

[B14] Cöllen E. Tanaskov Y. Holzer A. K. Dipalo M. Schafer J. Kraushaar U. (2024). Elements and development processes for test methods in toxicology and human health-relevant life science research. ALTEX 41 (1), 142–148. 10.14573/altex.2401041 38207287

[B15] Cosnier F. Seidel C. Valentino S. Schmid O. Bau S. Vogel U. (2021). Retained particle surface area dose drives inflammation in rat lungs following acute, subacute, and subchronic inhalation of nanomaterials. Part Fibre Toxicol. 18 (1), 29. 10.1186/s12989-021-00419-w 34353337 PMC8340536

[B16] David C. Brea D. Verney C. Cezard A. Vasseur V. Briard B. (2025). A comprehensive evaluation of murine and human *ex vivo* cultured alveolar macrophages. ERJ Open Res. 11 (3), 00859–02024. 10.1183/23120541.00859-2024 40391064 PMC12086855

[B17] DGUV (2020). Grenzwerte Staub: stoffspezifische Regelungen und ASGW als allgemeine Obergrenze; Hinweise zu Stäuben mit spezifischer Toxizität. Available online at: https://www.dguv.de/staub-info/rechtsgrundlagen/grenzwerte/index.jsp (Accessed March 15, 2026).

[B18] DGUV (2023). Allgemeiner Staubgrenzwert (ASGW): Anwendung Und Geltungsbereich (TRGS 900). Available online at: https://www.dguv.de/staub-info/rechtsgrundlagen/grenzwerte/asgw/index.jsp (Accessed March 15, 2026).

[B19] Donaldson K. Seaton A. (2012). A short history of the toxicology of inhaled particles. Part Fibre Toxicol. 9, 13. 10.1186/1743-8977-9-13 22559156 PMC3436721

[B20] Donaldson K. Wallace W. Elliott T. Henry C. (2017). James craufurd gregory, 19Th century Scottish physicians, and the link between occupation as a coal miner and lung disease. J. R. Coll. Physicians Edinb. 47 (3), 296–302. 10.4997/jrcpe.2017.317 29465110

[B21] Driscoll K. E. (2022). Review of lung particle overload, rat lung cancer, and the conclusions of the Edinburgh expert panel-it’s time to revisit cancer hazard classifications for titanium dioxide and carbon black. Front. Public Health 10, 907318. 10.3389/fpubh.2022.907318 35968415 PMC9366718

[B22] Driscoll K. E. Borm P. J. A. (2020). Expert workshop on the hazards and risks of poorly soluble low toxicity particles. Inhal. Toxicol. 32 (2), 53–62. 10.1080/08958378.2020.1735581 32149535

[B23] Dunn P. K. Smyth G. K. (1996). Randomized quantile residuals. J. Comput. Graph. Statistics 5 (3), 236–244. 10.1080/10618600.1996.10474708

[B24] ECETOC (2013). Poorly Soluble Particles/Lung Overload. Technical Report No. 122”. (Brussels: European Centre for Ecotoxicology and Toxicology of Chemicals).

[B25] European Commission (2014). “Horizon 2020 work programme 2014–2015: general annexes. G. technology readiness levels (TRL),” in Extract from Part 19 - Commission Decision C(2014)4995.

[B26] European Commission (2025). Article 25 of Regulation (EC) No 1907/2006 of the European Parliament and of the Council of 18 December 2006 Concerning the Registration, Evaluation, Authorisation and Restriction of Chemicals (REACH), Establishing a European Chemicals Agency, Amending Directive 1999/45/EC and Repealing Council Regulation (EEC) No 793/93 and Commission Regulation (EC) No 1488/94 as Well as Council Directive 76/769/EEC and Commission Directives 91/155/EEC, 93/67/EEC, 93/105/EC and 2000/21/EC”, In: 02006R1907.

[B27] European Committee for Standardization (1993). Workplace Atmospheres - Size Fraction Definitions for Measurement of Airborne Particles.

[B28] Gottardo S. Alessandrelli M. Valeria A. Atluri R. Barberio G. Bekker C. (2017). Nanoreg Framework for the Safety Assessment of Nanomaterials. (eds.) Gottardo S. Crutzen H. Jantunen A.

[B29] Haber L. T. Bradley M. A. Buerger A. N. Behrsing H. Burla S. Clapp P. W. (2024). New approach methodologies (NAMs) for the *in vitro* assessment of cleaning products for respiratory irritation: workshop report. Front. Toxicol. 6, 1431790. 10.3389/ftox.2024.1431790 39439531 PMC11493779

[B30] Hartig F. (2024). DHARMa: residual diagnostics for hierarchical (Multi-Level/mixed) regression models. R. Package Version.0.4.7. 10.32614/CRAN.package.DHARMa

[B31] Hartwig A. MAK Commission (2020). α-Aluminium oxide (corundum) (respirable fraction). MAK value documentation, supplement- translation of the German version from 2019. MAK Collect. Occup. Health Saf. 5 (3). 10.34865/mb742990vere5_3ad

[B32] Jacobs M. N. Hoffmann S. Hollnagel H. M. Kern P. Kolle S. N. Natsch A. (2024). Avoiding a reproducibility crisis in regulatory toxicology-on the fundamental role of ring trials. Arch. Toxicol. 98 (7), 2047–2063. 10.1007/s00204-024-03736-z 38689008 PMC11169035

[B33] Janer G. Ag-Seleci D. Sergent J. A. Landsiedel R. Wohlleben W. (2021a). Creating sets of similar nanoforms with the ECETOC NanoApp: real-life case studies. Nanotoxicology 15 (8), 1016–1034. 10.1080/17435390.2021.1946186 34242099

[B34] Janer G. Landsiedel R. Wohlleben W. (2021b). Rationale and decision rules behind the ECETOC NanoApp to support registration of sets of similar nanoforms within REACH. Nanotoxicology 15 (2), 145–166. 10.1080/17435390.2020.1842933 33320695

[B35] Jensen K. A. Kembouche Y. Christiansen E. Jacobsen N. R. Wallin H. (2011). “Final protocol for producing suitable manufactured nanomaterial exposure media: the generic NANOGENOTOX dispersion protocol,” in Towards a Method for Detecting the Potential Genotoxicity of Nanomaterials the National Research Centre for the Working Environment.

[B36] Keller J. G. Graham U. M. Koltermann-Jully J. Gelein R. Ma-Hock L. Landsiedel R. (2020). Predicting dissolution and transformation of inhaled nanoparticles in the lung using abiotic flow cells: the case of barium sulfate. Sci. Rep. 10 (1), 458. 10.1038/s41598-019-56872-3 31949204 PMC6965653

[B37] Kelsall J. Hoffman E. Tha E. Ma-Hock L. Landsiedel R. Hutter V. (2025). A comparison between in vitro responses of human alveolar macrophage-like cells (ImmuPHAGE™) and rat alveolar macrophages (NR8383) for predicting short-term inhalation toxicity of particles. The Toxicologist, Supplement to Toxicological Sciences 204 (S1).

[B38] Klein C. L. Wiench K. Wiemann M. Ma-Hock L. van Ravenzwaay B. Landsiedel R. (2012). Hazard identification of inhaled nanomaterials: making use of short-term inhalation studies. Arch. Toxicol. 86 (7), 1137–1151. 10.1007/s00204-012-0834-2 22532024

[B39] Koltermann-Jülly J. Keller J. G. Vennemann A. Werle K. Müller P. Ma-Hock L. (2018). Abiotic dissolution rates of 24 (nano)forms of 6 substances compared to macrophage-assisted dissolution and *in vivo* pulmonary clearance: grouping by biodissolution and transformation. NanoImpact 12, 29–41. 10.1016/j.impact.2018.08.005

[B40] Konduru N. Keller J. Ma-Hock L. Groters S. Landsiedel R. Donaghey T. C. (2014). Biokinetics and effects of barium sulfate nanoparticles. Part Fibre Toxicol. 11, 55. 10.1186/s12989-014-0055-3 25331813 PMC4219084

[B41] Krombach F. Munzing S. Allmeling A. M. Gerlach J. T. Behr J. Dorger M. (1997). Cell size of alveolar macrophages: an interspecies comparison. Environ. Health Perspect. 105 (Suppl. 5), 1261–1263. 10.1289/ehp.97105s51261 9400735 PMC1470168

[B42] Landsiedel R. Ma-Hock L. Hofmann T. Wiemann M. Strauss V. Treumann S. (2014). Application of short-term inhalation studies to assess the inhalation toxicity of nanomaterials. Part Fibre Toxicol. 11, 16. 10.1186/1743-8977-11-16 24708749 PMC4113196

[B43] Landsiedel R. Birk B. Demuth P. Fabian E. Hewitt N. J. Hollnagel H. M. (2024). The use of toxicokinetic information for setting concentrations of *in vitro* toxicity tests and for interpreting their results: a proposed workflow. Appl. Vitro Toxicol. 10 (1), 15–26. 10.1089/aivt.2023.0018

[B44] Lenth R. V. Piaskowski J. (2025). Emmeans: estimated marginal means, aka least-squares means. R package version 2.0.1. 10.32614/CRAN.package.emmeans

[B45] Li T. Yu Y. Sun Z. Duan J. (2022). A comprehensive understanding of ambient particulate matter and its components on the adverse health effects based from epidemiological and laboratory evidence. Part Fibre Toxicol. 19 (1), 67. 10.1186/s12989-022-00507-5 36447278 PMC9707232

[B46] Liu Q. Yang L. Li C. Han L. Wilmot J. Yang G. (2025). Alveolar macrophages initiate the spatially targeted recruitment of neutrophils after nanoparticle inhalation. Sci. Adv. 11 (45), eadx8586. 10.1126/sciadv.adx8586 41202119 PMC12594175

[B47] Lüdecke D. Ben-Shachar M. S. Patil I. Waggoner P. Makowski D. (2021). Performance: an R package for assessment, comparison and testing of statistical models. J. Open Source Softw. 6 (60), 3139. 10.21105/joss.03139

[B48] Ma-Hock L. Sauer U. G. Ruggiero E. Keller J. G. Wohlleben W. Landsiedel R. (2021). The use of nanomaterial *in vivo* organ burden data for *in vitro* dose setting. Small 17 (15), e2005725. 10.1002/smll.202005725 33586349

[B49] Ma-Hock L. Stratmann H. Hufnagel M. Bowden A. M. Serrano Ramon B. Warheit D. (2026). A quantitative definition for poorly soluble particles. Part Fibre Toxicol. 23 (1), 13. 10.1186/s12989-026-00670-z 41821001 PMC12983584

[B50] Molina R. M. Konduru N. V. Queiroz P. M. Figueroa B. Fu D. Ma-Hock L. (2019). Fate of barium sulfate nanoparticles deposited in the lungs of rats. Sci. Rep. 9 (1), 8163. 10.1038/s41598-019-44551-2 31160608 PMC6546789

[B51] Morrow P. E. (1988). Possible mechanisms to explain dust overloading of the lungs. Fundam. Appl. Toxicol. 10 (3), 369–384. 10.1016/0272-0590(88)90284-9 3286345

[B52] Morrow P. E. (1992). Dust overloading of the lungs: update and appraisal. Toxicol. Appl. Pharmacol. 113 (1), 1–12. 10.1016/0041-008X(92)90002-A 1553742

[B53] NASA (2017). Technology Readiness Level Definitions. Washington (DC): National Aeronautics and Space Administration.

[B54] Oberdörster G. (1988). Lung clearance of inhaled insoluble and soluble particles. J. Aerosol Med. 1 (4), 289–330. 10.1089/jam.1988.1.289

[B55] Oberdörster G. (1996). Significance of particle parameters in the evaluation of exposure-dose-response relationships of inhaled particles. Particul. Sci. Technol. 14 (2), 131–151. 10.1080/02726359608906690 11542496

[B56] Oberdörster G. Ferin J. Gelein R. Soderholm S. C. Finkelstein J. (1992). Role of the alveolar macrophage in lung injury: studies with ultrafine particles. Environ. Health Perspect. 97, 193–199. 10.1289/ehp.97-1519541 1396458 PMC1519541

[B57] Oberdörster G. Oberdörster E. Oberdörster J. (2005). Nanotoxicology: an emerging discipline evolving from studies of ultrafine particles. Environ. Health Perspect. 113 (7), 823–839. 10.1289/ehp.7339 16002369 PMC1257642

[B58] OECD (2018). Guidance Document on Good in Vitro Method Practices (GIVIMP) in OECD Series on Testing and Assessment (Paris).

[B59] Padmore T. Stark C. Turkevich L. A. Champion J. A. (2017). Quantitative analysis of the role of fiber length on phagocytosis and inflammatory response by alveolar macrophages. Biochim. Biophys. Acta Gen. Subj. 1861 (2), 58–67. 10.1016/j.bbagen.2016.09.031 27784615 PMC5228597

[B60] Pauluhn J. (2009). Pulmonary toxicity and fate of agglomerated 10 and 40 nm aluminum oxyhydroxides following 4-week inhalation exposure of rats: toxic effects are determined by agglomerated, not primary particle size. Toxicol. Sci. 109 (1), 152–167. 10.1093/toxsci/kfp046 19251949

[B61] Pauluhn J. (2011). Poorly soluble particulates: searching for a unifying denominator of nanoparticles and fine particles for DNEL estimation. Toxicology 279, 1879–3185. 10.1016/j.tox.2010.10.009 21074595

[B62] Pauluhn J. (2014). Derivation of occupational exposure levels (OELs) of low-toxicity isometric biopersistent particles: how can the kinetic lung overload paradigm be used for improved inhalation toxicity study design and OEL-derivation? Part Fibre Toxicol. 11, 72. 10.1186/s12989-014-0072-2 25526747 PMC4323034

[B63] R Core Team (2025). R: A Language and Environment for Statistical Computing. Vienna, Austria: R Foundation for Statistical Computing.

[B64] Rehn B. Rehn S. Bruch J. (1999). Ein neues in vitro-Prüfkonzept (Vektorenmodell) zum biologischen Screening und Monitoring der Lungentoxicität von Stäuben. Gefahrst. - Reinhalt. Luft 59, 181–188.

[B65] Riediker M. Zink D. Kreyling W. Oberdörster G. Elder A. Graham U. (2019). Particle toxicology and health - where are we? Part Fibre Toxicol. 16 (1), 19. 10.1186/s12989-019-0302-8 31014371 PMC6480662

[B66] Ritz C. Baty F. Streibig J. C. Gerhard D. (2016). Dose-response analysis using R. PLOS ONE 10 (12), e0146021. 10.1371/journal.pone.0146021 26717316 PMC4696819

[B67] Rosolowski J. Weber T. Malakpour-Permlid A. Oredsson S. (2025). Revisiting 3Rs: rethinking replacement and new approach methodologies. Front. Toxicol. 7, 1664209. 10.3389/ftox.2025.1664209 41322701 PMC12660069

[B68] Sager T. M. Kommineni C. Castranova V. (2008). Pulmonary response to intratracheal instillation of ultrafine versus fine titanium dioxide: role of particle surface area. Part Fibre Toxicol. 5, 17. 10.1186/1743-8977-5-17 19046442 PMC2633346

[B69] Schinke M. Nguyen A. H. H. Náger T. Abdin S. Gensch I. Ackermann M. (2025). P30-34 scalable generation and use of human alveolar-like macrophages to standardize inhalation toxicology and pulmonary immune response. Toxicol. Lett. 411, S389–S389a. 10.1016/j.toxlet.2025.07.895

[B70] Schmid O. Stoeger T. (2016). Surface area is the biologically most effective dose metric for acute nanoparticle toxicity in the lung. J. Aerosol Sci. 99, 133–143. 10.1016/j.jaerosci.2015.12.006

[B71] Schmid O. Moller W. Semmler-Behnke M. Ferron G. A. Karg E. Lipka J. (2009). Dosimetry and toxicology of inhaled ultrafine particles. Biomarkers 14 (Suppl. 1), 67–73. 10.1080/13547500902965617 19604063

[B72] Scott L. Kelsall J. Tha E. Ma-Hock L. Landsiedel R. Hutter V. (2025). P17-07 *in vitro* approaches for inhalation safety assessment of poorly soluble and insoluble materials. Toxicol. Lett. 411, S180–S181. 10.1016/j.toxlet.2025.07.435

[B73] Shan Q. Dong Z. Li N. Zhou Z. Ren J. Liu W. (2026). Deciphering the heterogeneity of pulmonary macrophages in response to fine particles. Small 22 (10), e07293. 10.1002/smll.202507293 41504080 PMC12910429

[B74] Stratmann H. Wohlleben W. Wiemann M. Vennemann A. End N. Veith U. (2021). Classes of organic pigments meet tentative PSLT criteria and lack toxicity in short-term inhalation studies. Regul. Toxicol. Pharmacol. 124, 104988. 10.1016/j.yrtph.2021.104988 34224799

[B75] Stucki A. O. Barton-Maclaren T. S. Bhuller Y. Henriquez J. E. Henry T. R. Hirn C. (2022). Use of new approach methodologies (NAMs) to meet regulatory requirements for the assessment of industrial chemicals and pesticides for effects on human health. Front. Toxicol. 4, 964553. 10.3389/ftox.2022.964553 36119357 PMC9475191

[B76] Takebayashi T. Landsiedel R. Gamo M. (2019). in *In vivo* Inhalation Toxicity Screening Methods for Manufactured Nanomaterials (Singapore: Springer).

[B77] Thoma T. Ma-Hock L. Schneider S. Honarvar N. Treumann S. Groeters S. (2024). Toxicological inhalation studies in rats to substantiate grouping of zinc oxide nanoforms. Part Fibre Toxicol. 21 (1), 24. 10.1186/s12989-024-00572-y 38760761 PMC11100124

[B78] van der Valk J. Bieback K. Buta C. Cochrane B. Dirks W. G. Fu J. (2018). Fetal serum (FBS): past - present - future. ALTEX 35 (1), 99–118. 10.14573/altex.1705101 28800376

[B79] Verdanova M. Sauerova P. Hempel U. Kalbacova M. H. (2017). Initial cell adhesion of three cell types in the presence and absence of serum proteins. Histochem Cell Biol. 148 (3), 273–288. 10.1007/s00418-017-1571-7 28432431

[B80] Weber T. Malakpour-Permlid A. Chary A. D’Alessandro V. Haut L. Seufert S. (2025). Fetal bovine serum: how to leave it behind in the pursuit of more reliable science. Front. Toxicol. 7, 1612903. 10.3389/ftox.2025.1612903 40861932 PMC12371577

[B81] Wiemann M. Vennemann A. Sauer U. G. Wiench K. Ma-Hock L. Landsiedel R. (2016). An *in vitro* alveolar macrophage assay for predicting the short-term inhalation toxicity of nanomaterials. J. Nanobiotechnology 14, 16. 10.1186/s12951-016-0164-2 26944705 PMC4779246

[B82] Worth A. P. Balls M. (2001). The importance of the prediction model in the validation of alternative tests. Altern. Laboratory Animals 29 (2), 135–143. 10.1177/026119290102900210 11262759

[B83] Zenker F. A. (1867). Usher Staubinhalationskrankeheiten der Lungen. Dtsch. Arch. Klin. Med. 2, 116.

