# Supplementary material for: Readiness of the in vitro alveolar macrophage assay: an interlaboratory comparison using 12 blinded particles
Source: Front Toxicol. 2026 Aug 28;8:1844090. doi: 10.3389/ftox.2026.1844090 (PMC13561502; doi:10.3389/ftox.2026.1844090)

Supplementary Images
Particle uptake after 16 hours of exposure

Methods

Micrographs were taken using inverted phase-contrast microscopy from both cell-free particle controls and exposed cells at the lowest (2.8 or 22.5 µg/mL) and highest (22.5 or 180 µg/mL) concentrations for each particle after 16 hours of incubation. Particle numbers correspond to codes assigned during blinding.

Lead Laboratory
Particle 1 – TiO_2 NM-101

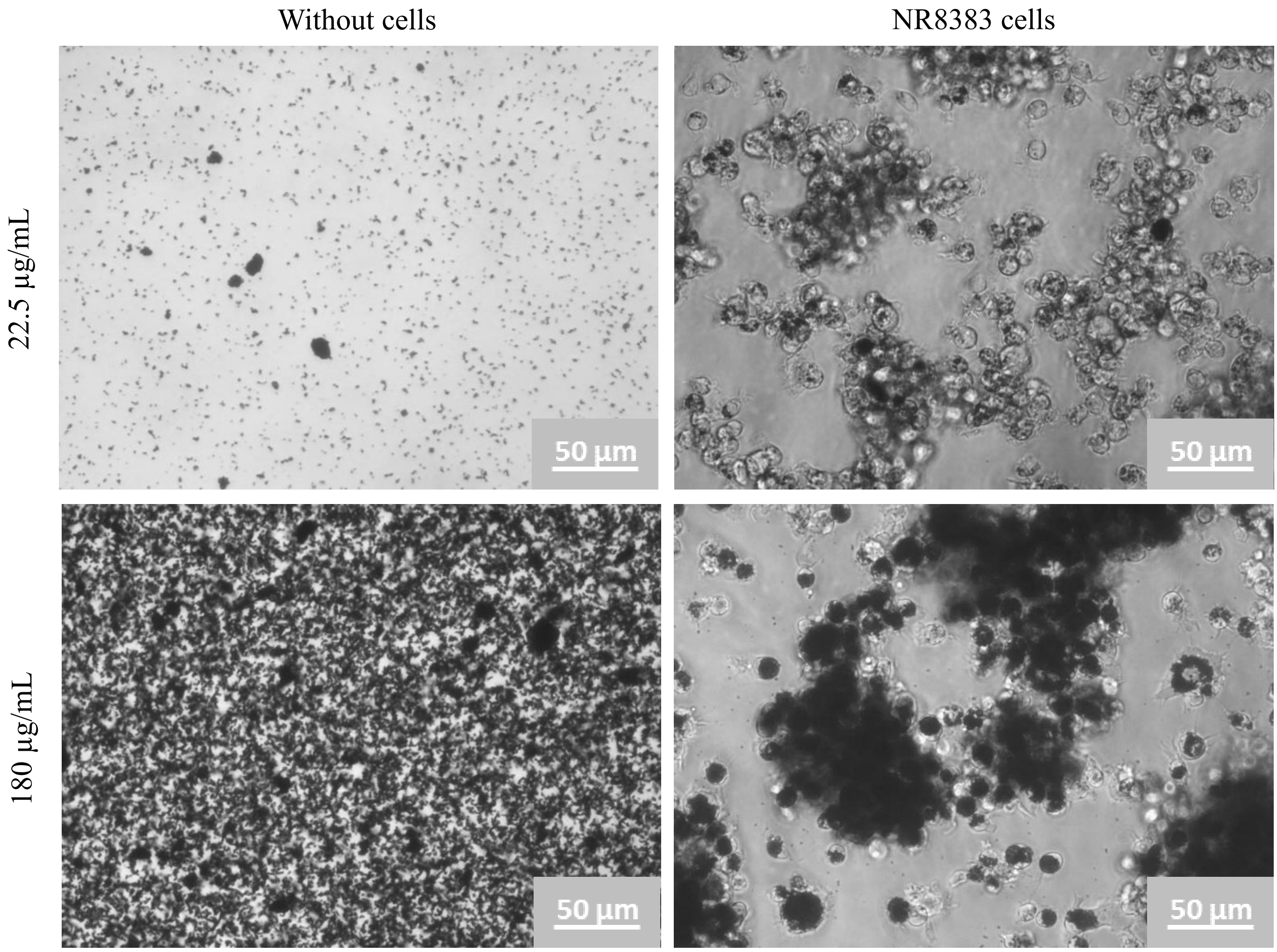

Lead Laboratory
Particle 2 – Mn_2O_3

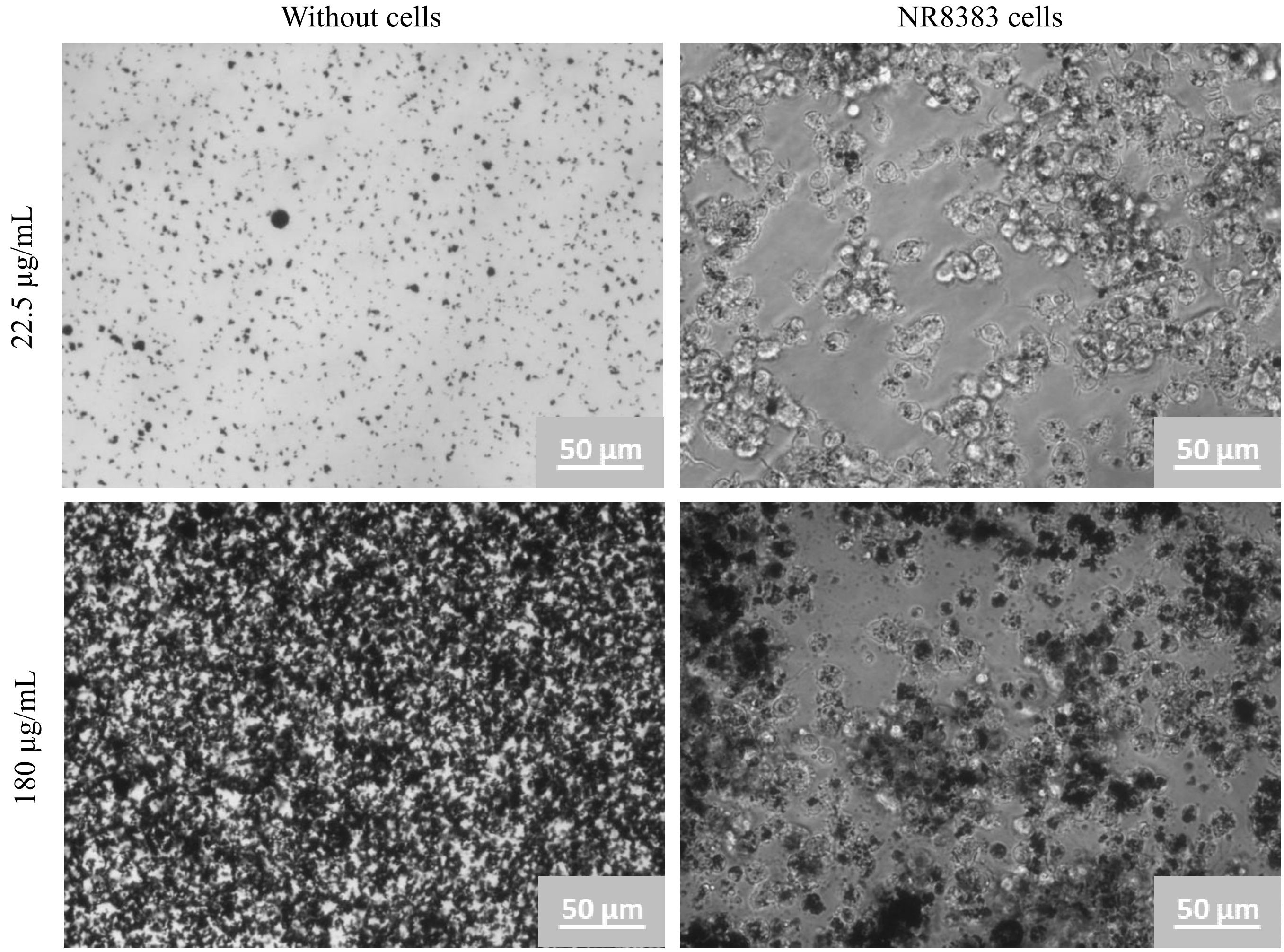

Lead Laboratory
Particle 3 – TiO_2 NM-105

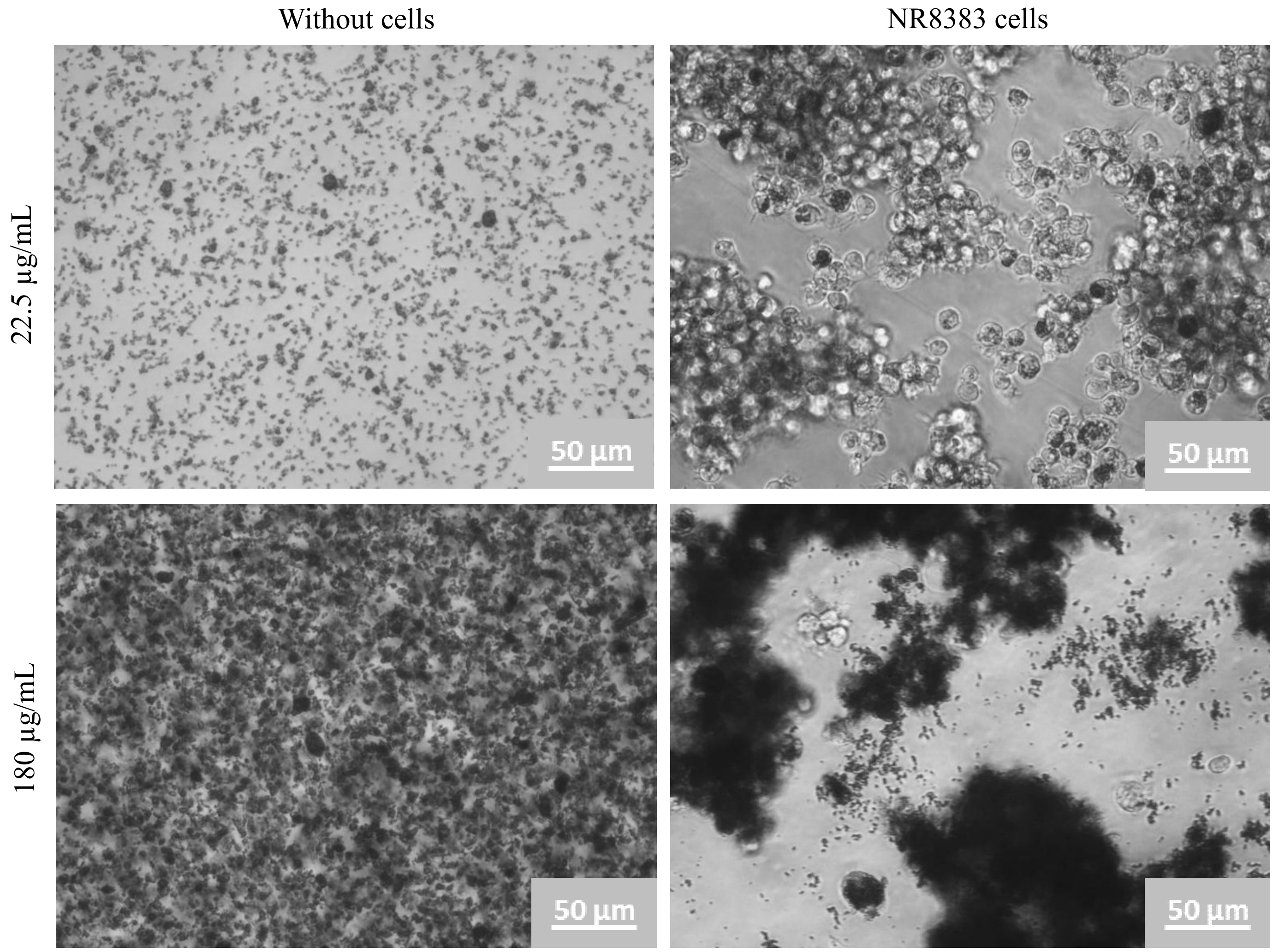

Lead Laboratory
Particle 4 – Tungsten carbide

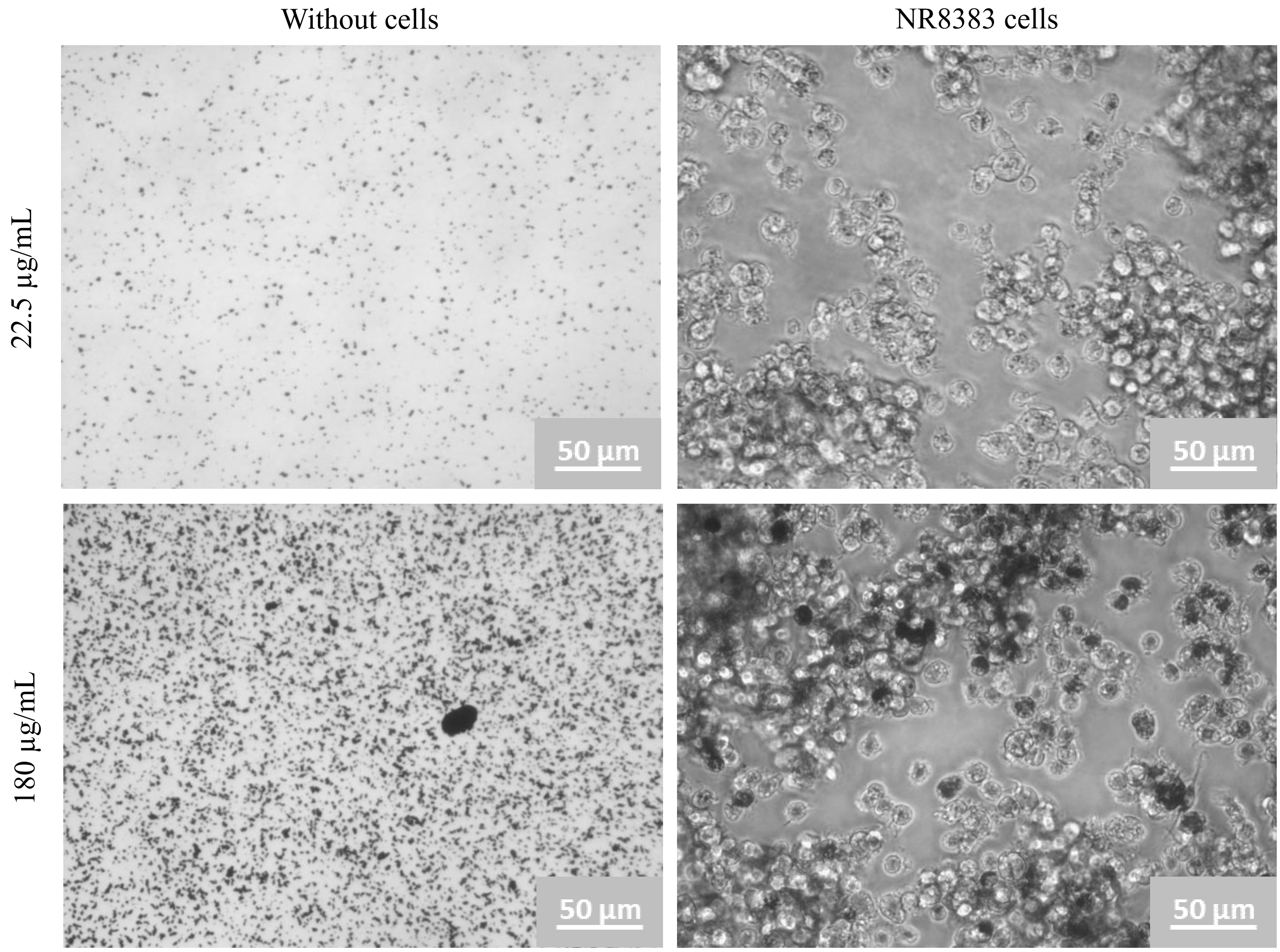

Lead Laboratory
Particle 5 – Tungsten carbide-Cobalt

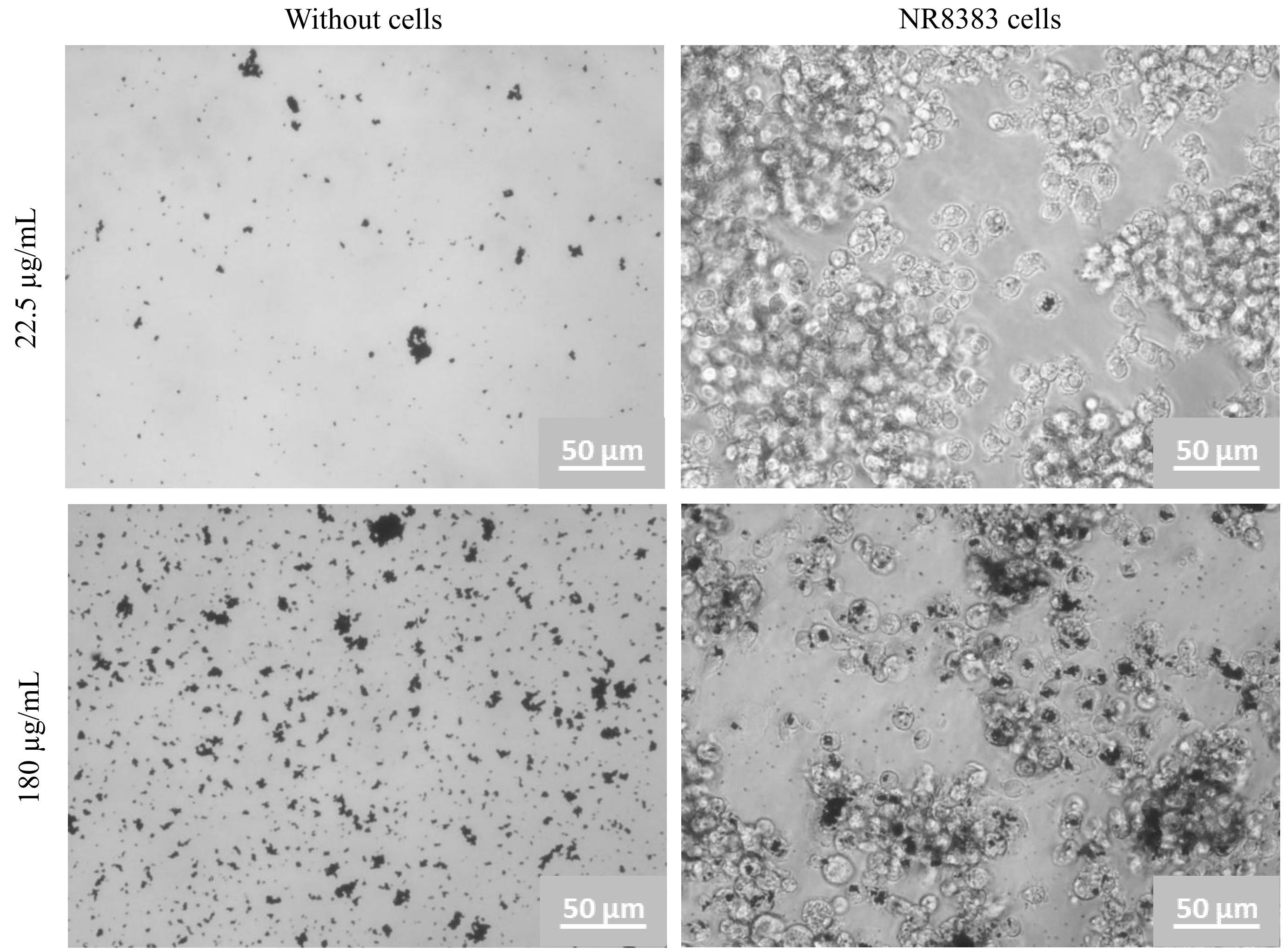

Lead Laboratory
Particle 6 – CeO_2 NM-212

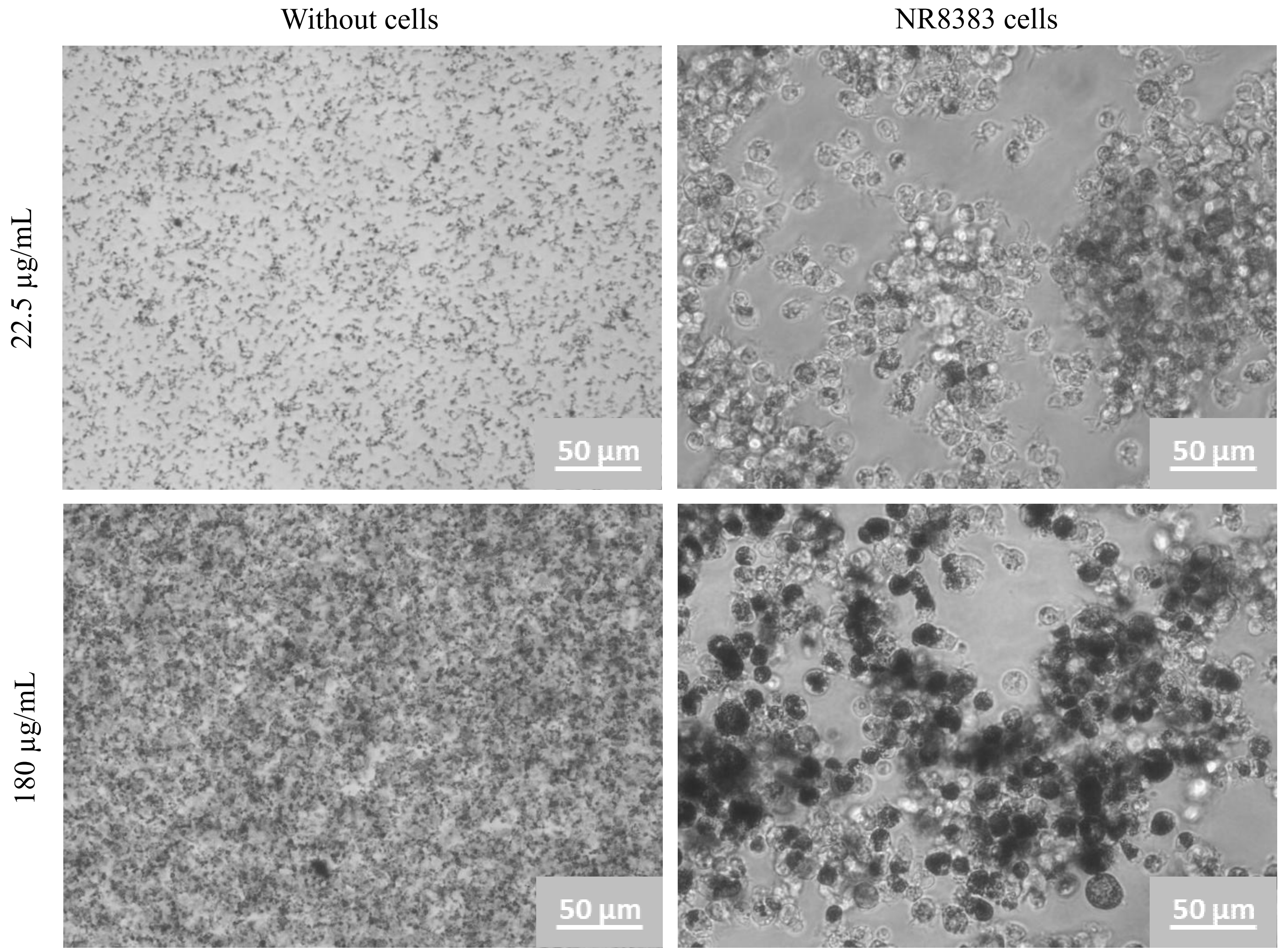

Lead Laboratory
Particle 7 – SiO_2 NM-203

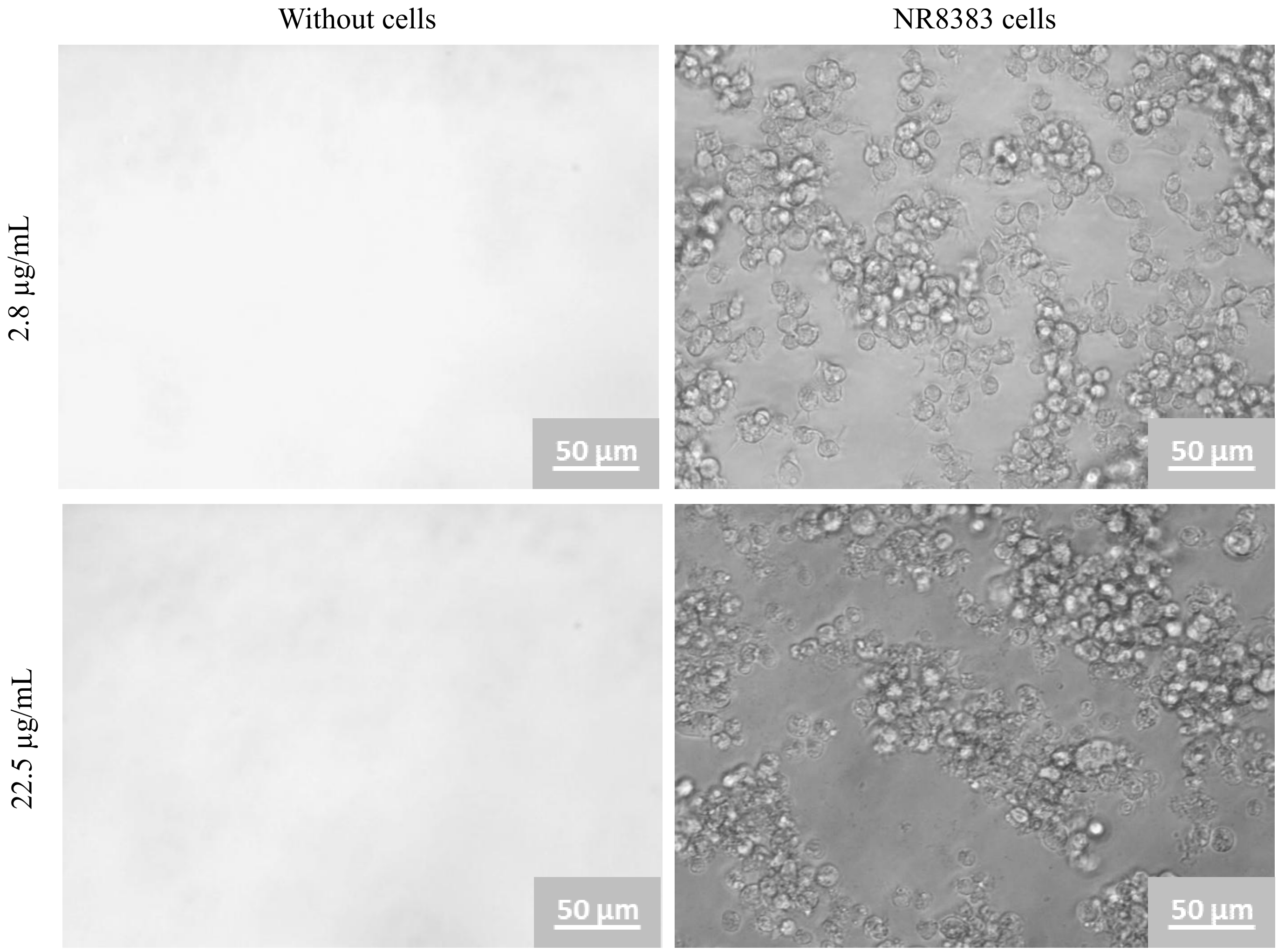

SiO_2 NM-203 were not visible in phase-contrast microscopy.

Lead Laboratory
Particle 8 – ZnO NM-111

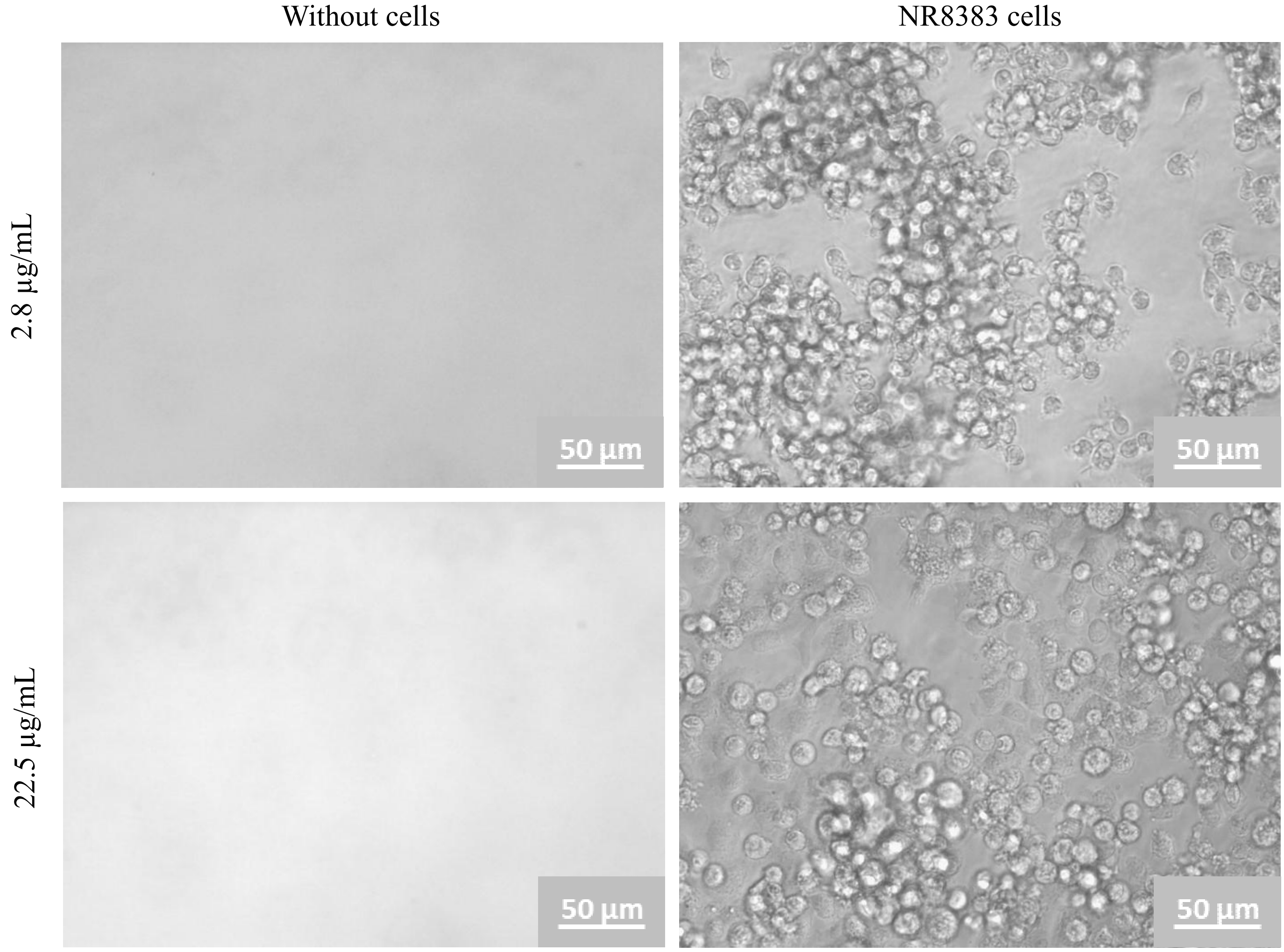

ZnO NM-111 were not visible in phase-contrast microscopy.

Lead Laboratory
Particle 9 – Quartz DQ12 nanowires

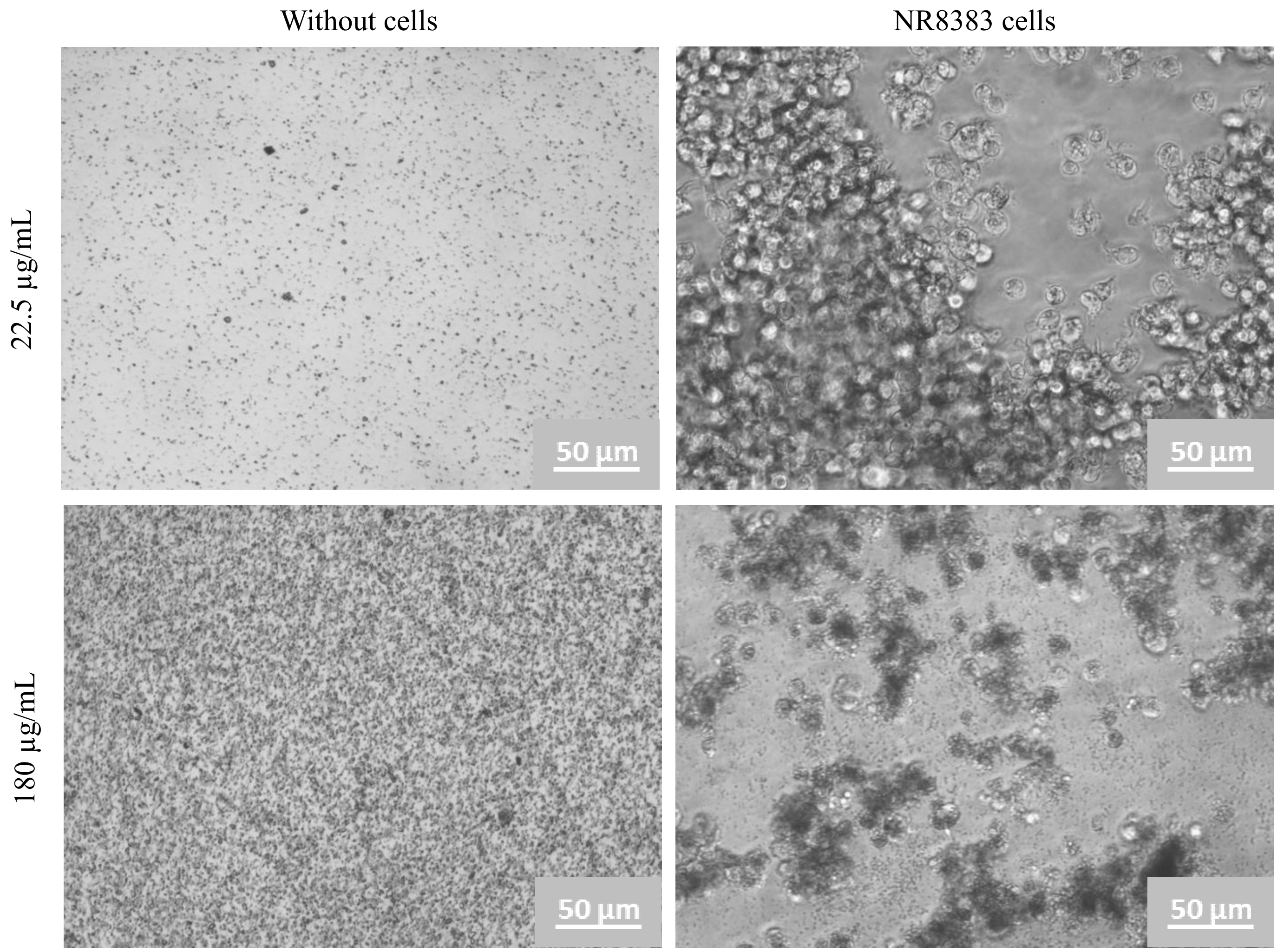

Lead Laboratory
Particle 10 – SiC nanowires

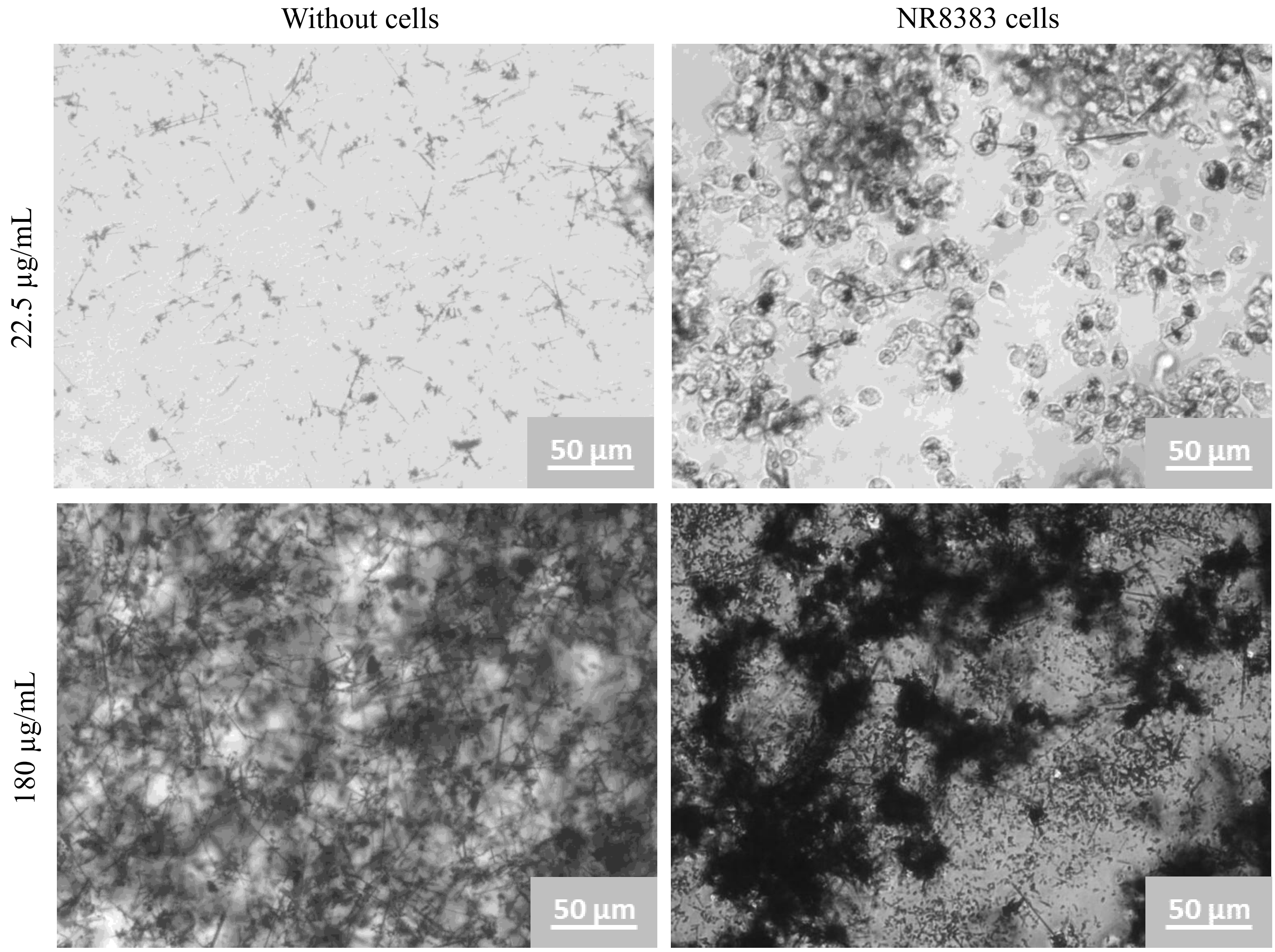

Lead Laboratory
Particle 11 – $BaSO_4$

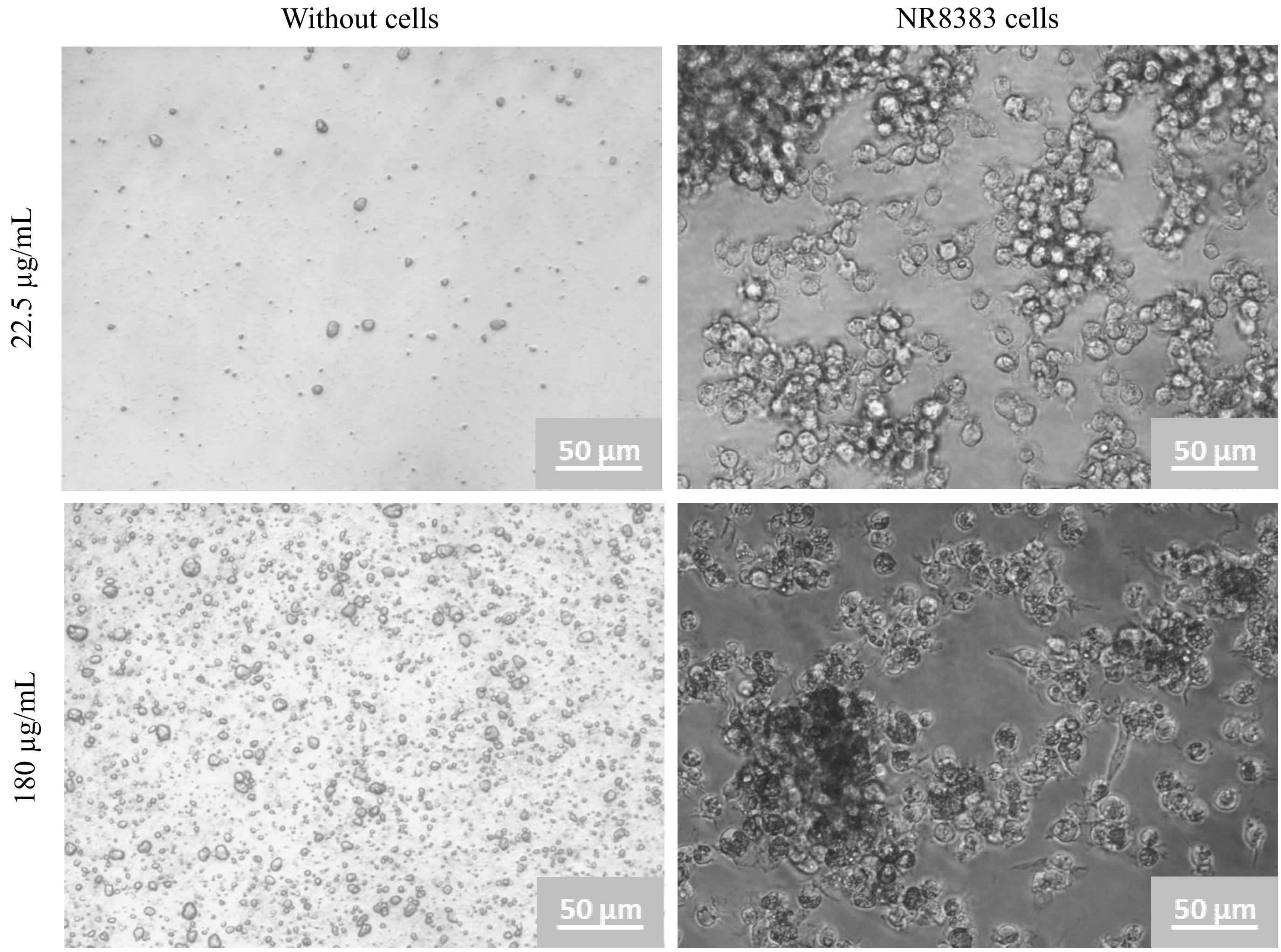

Lead Laboratory
Particle 12 – Corundum

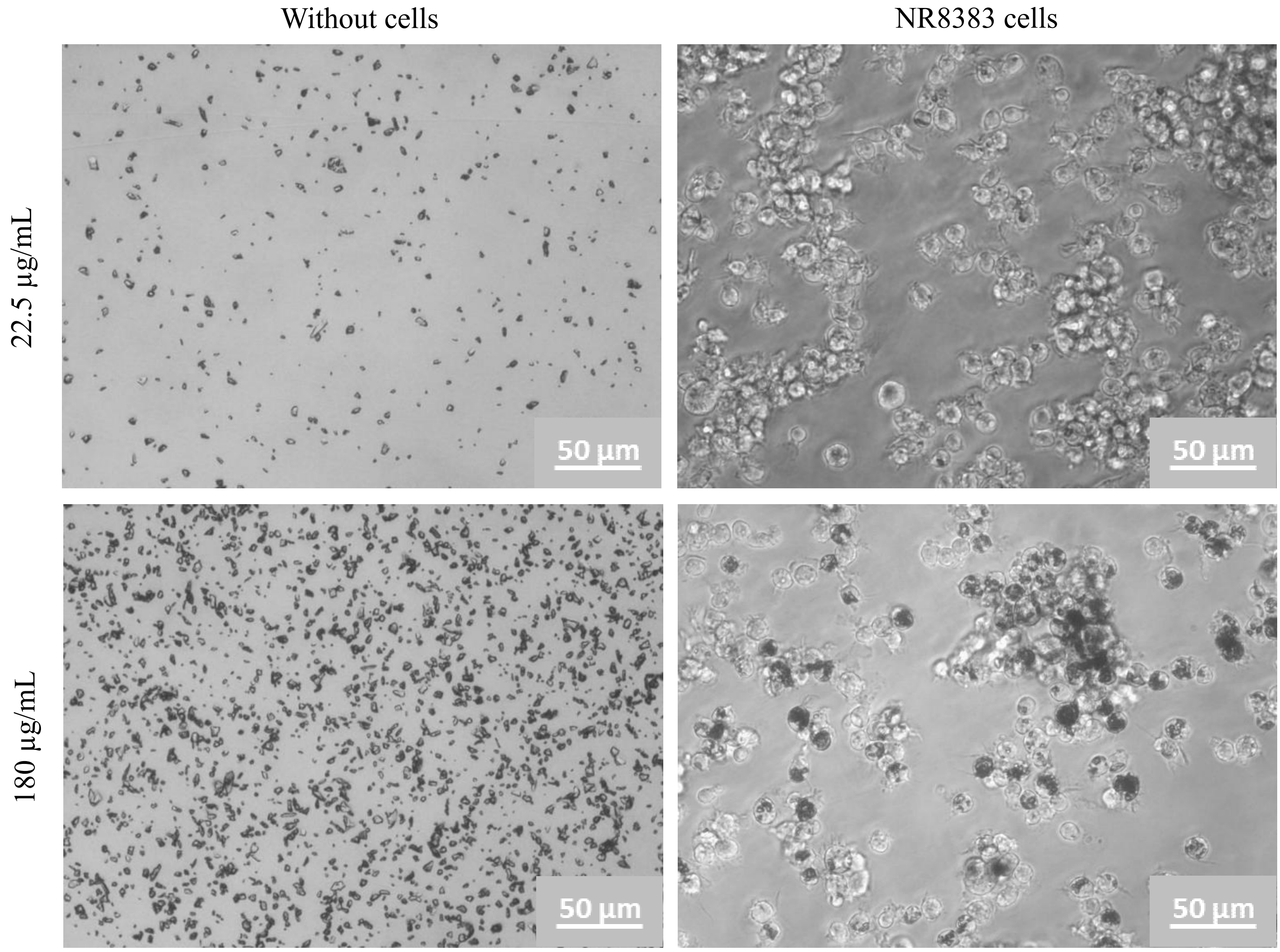

Lab1
Particle 1 – TiO_2 NM-101

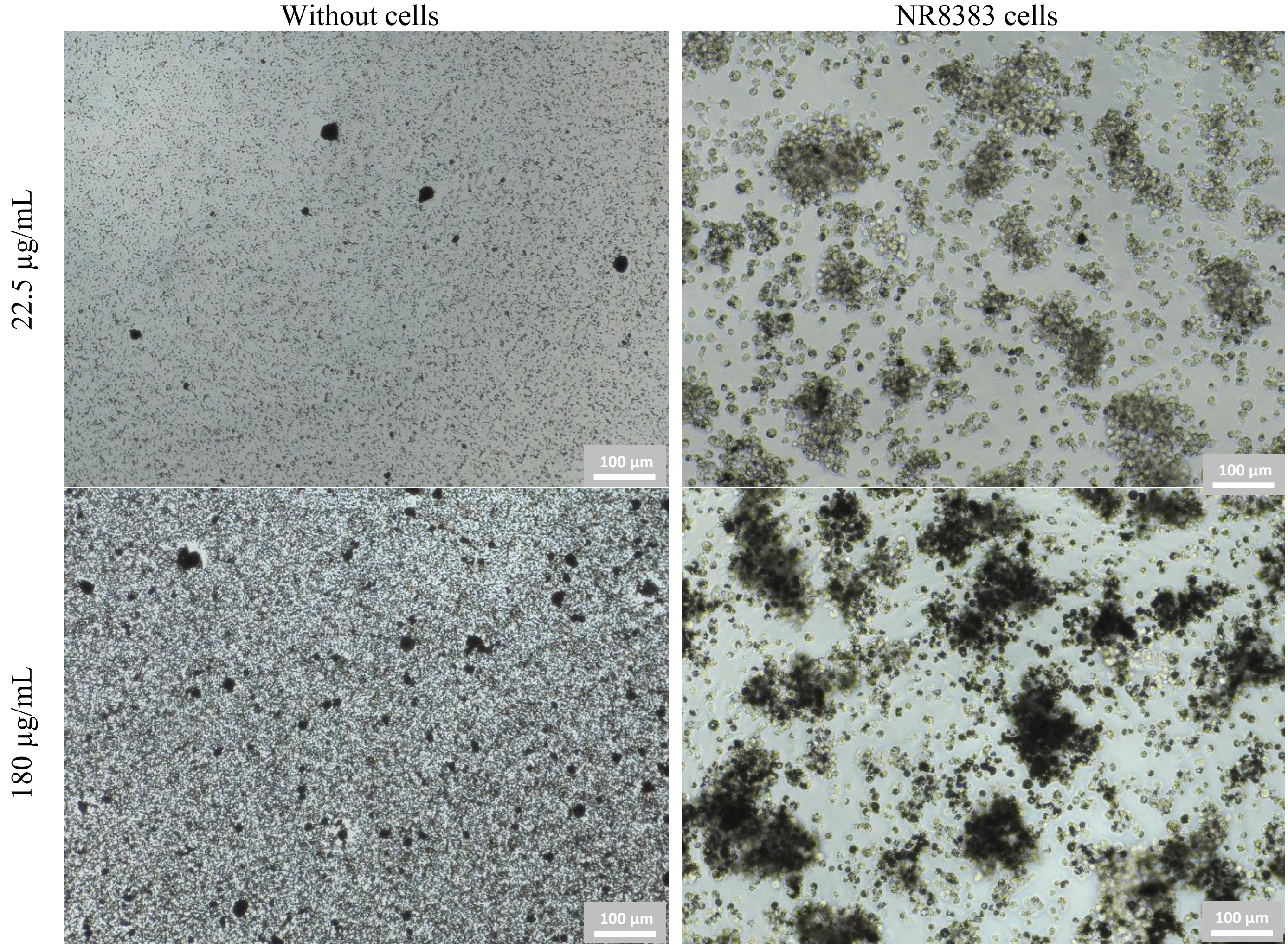

Lab1
Particle 2 – Mn_2O_3

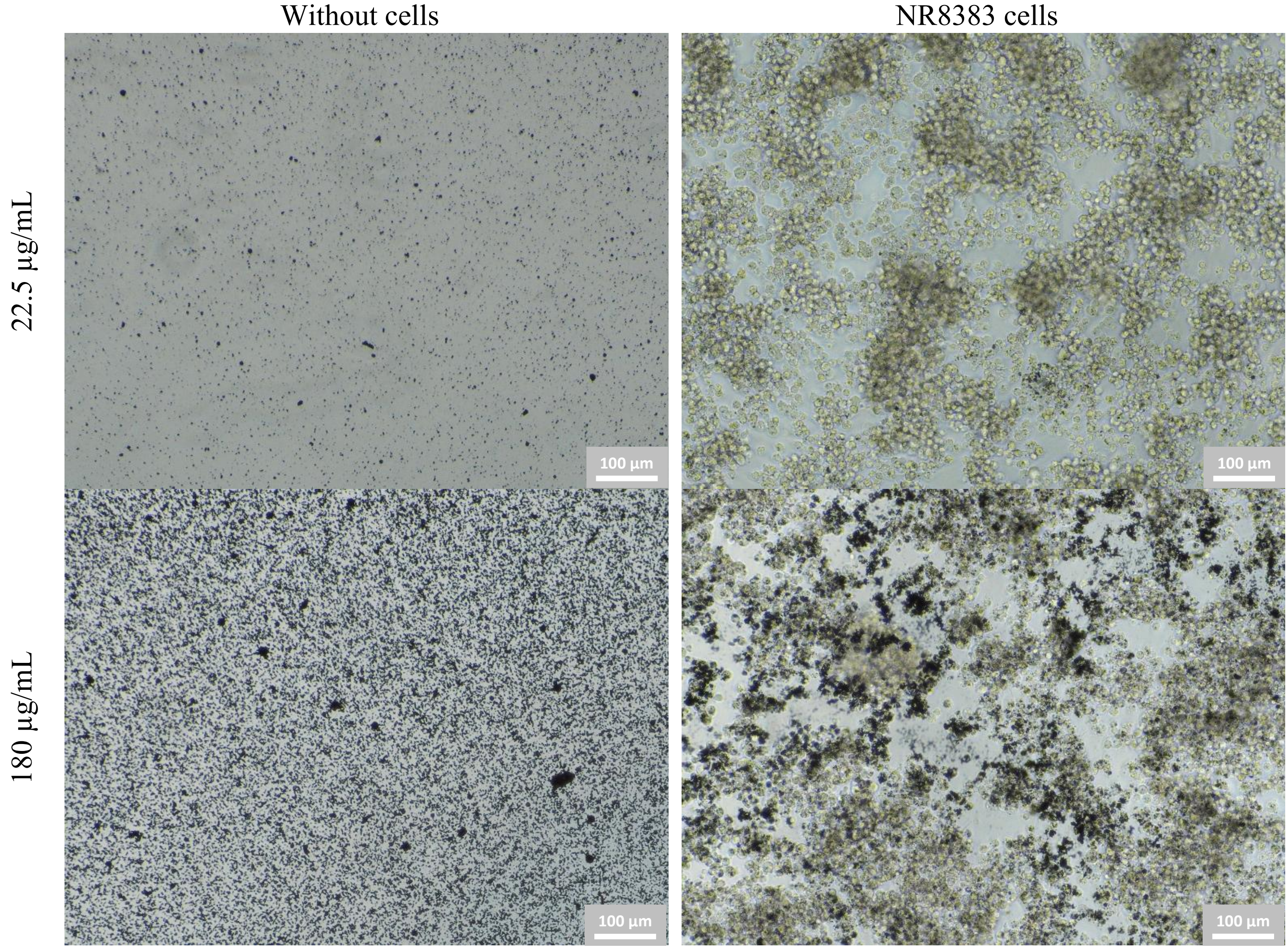

Lab1
Particle 3 - TiO2 NM-105

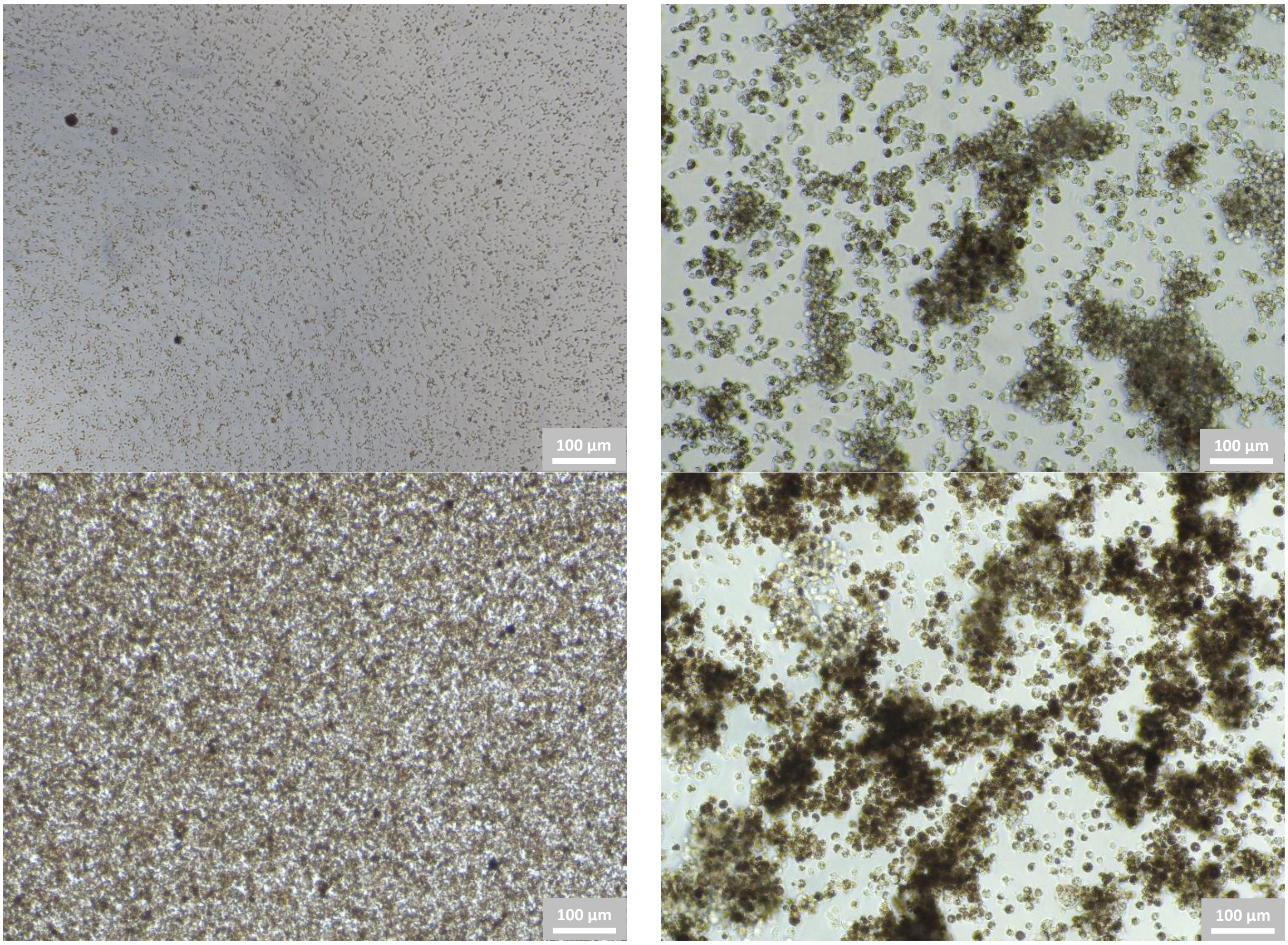

Lab1

Particle 4 - Tungsten carbide

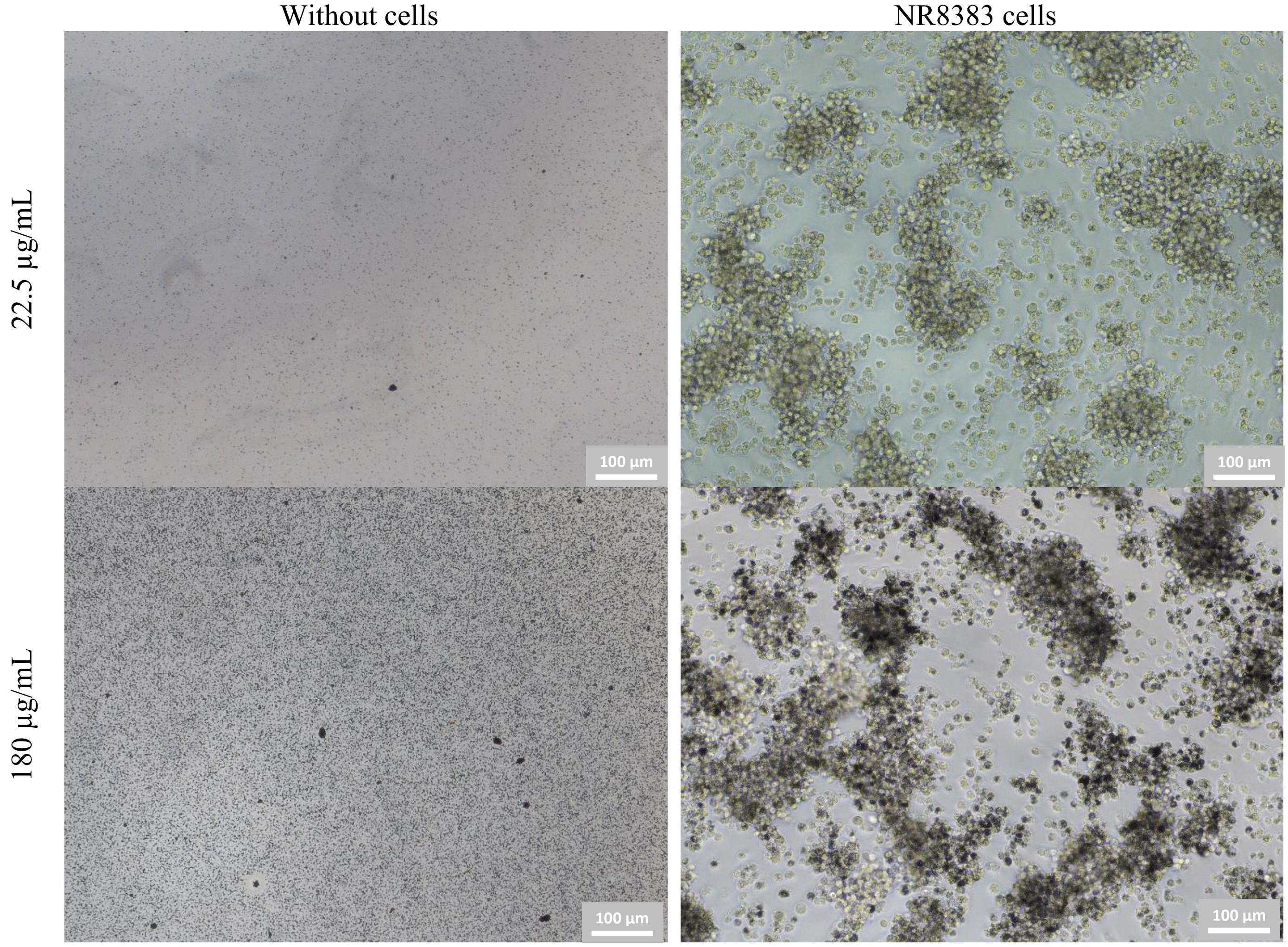

Lab 1
Particle 5 - Tungsten carbide-Cobalt

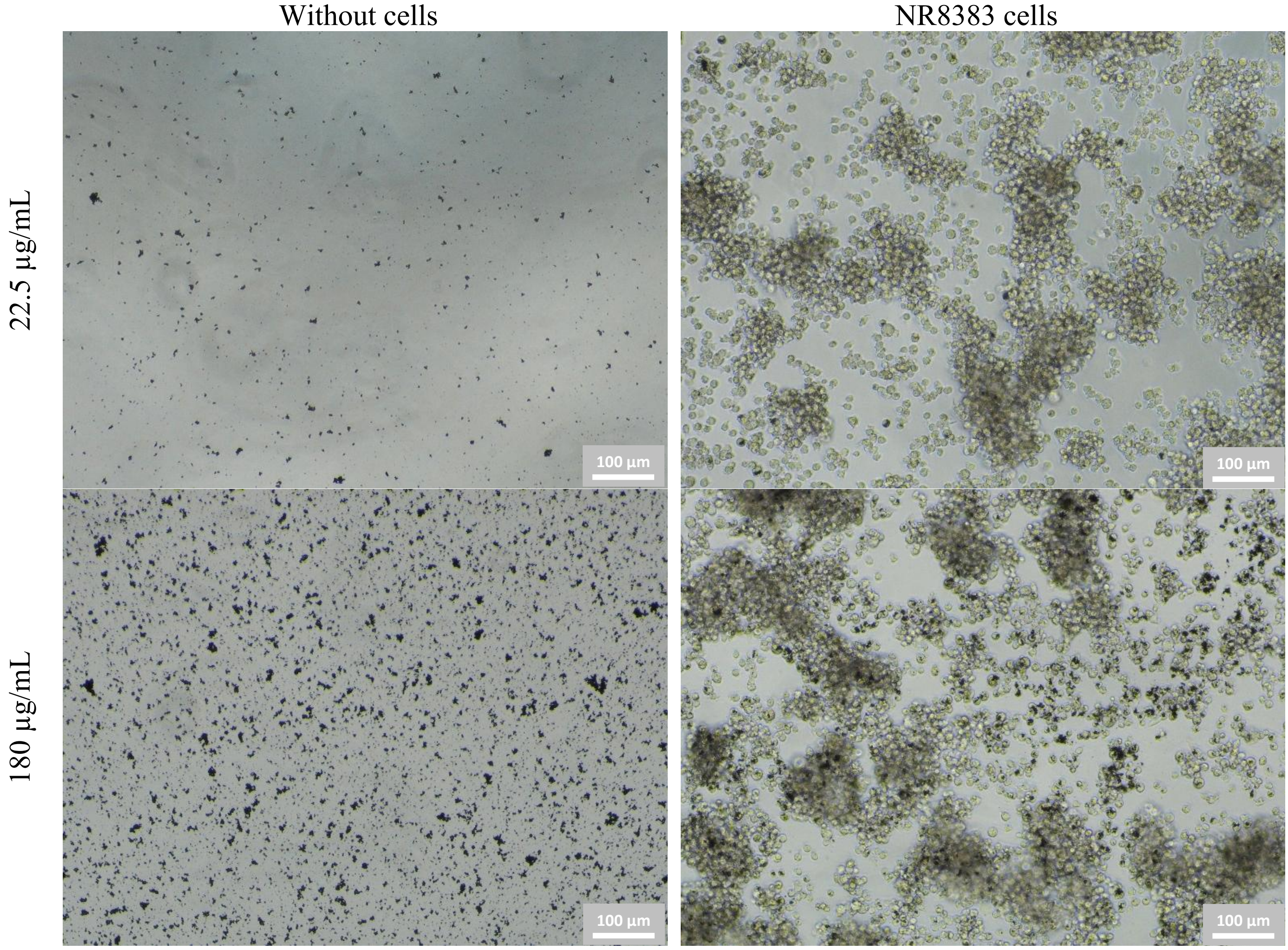

Lab1
Particle 6 - CeO_2 NM-212

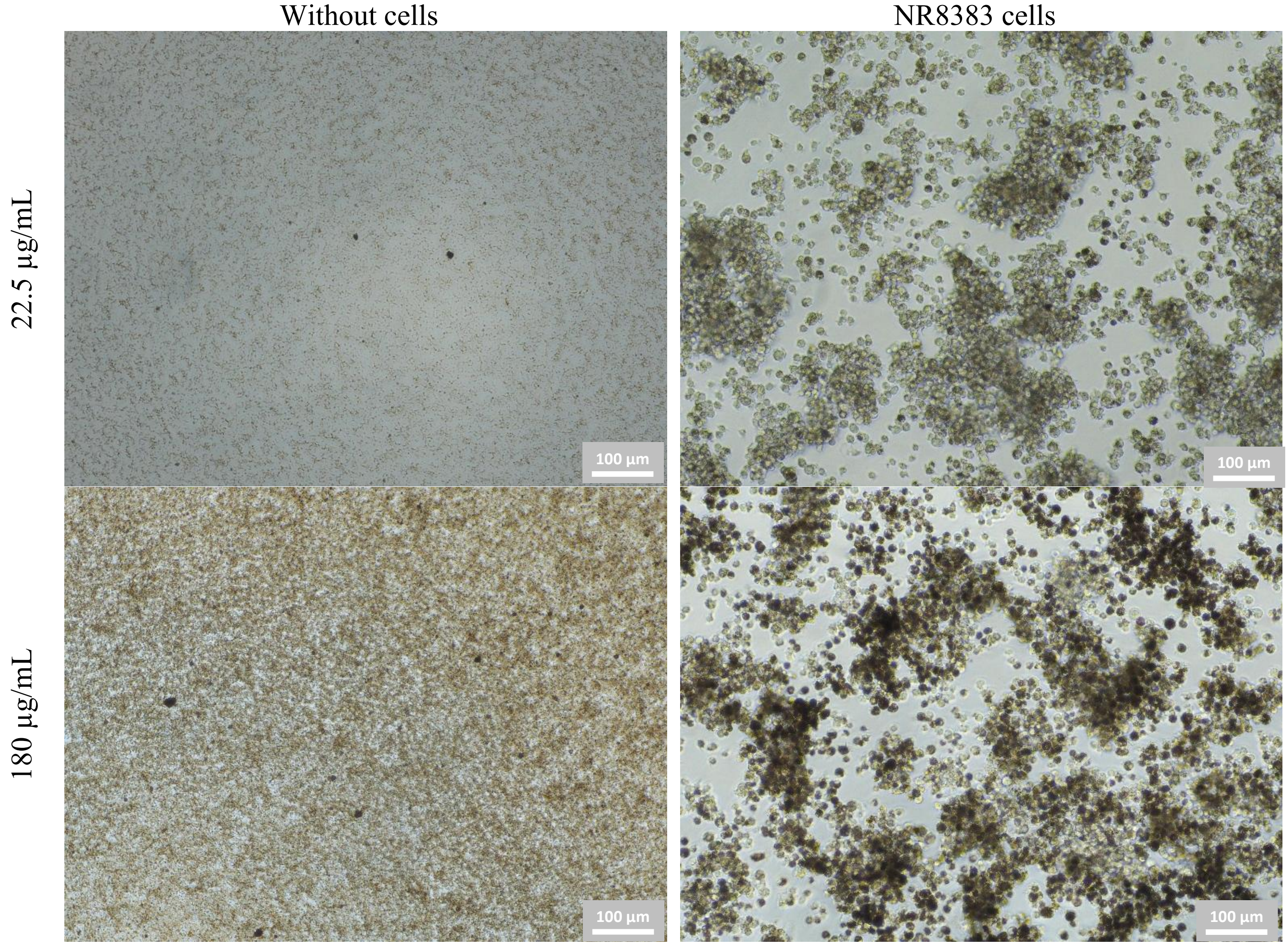

Lab1
Particle 7 - SiO2 NM-203

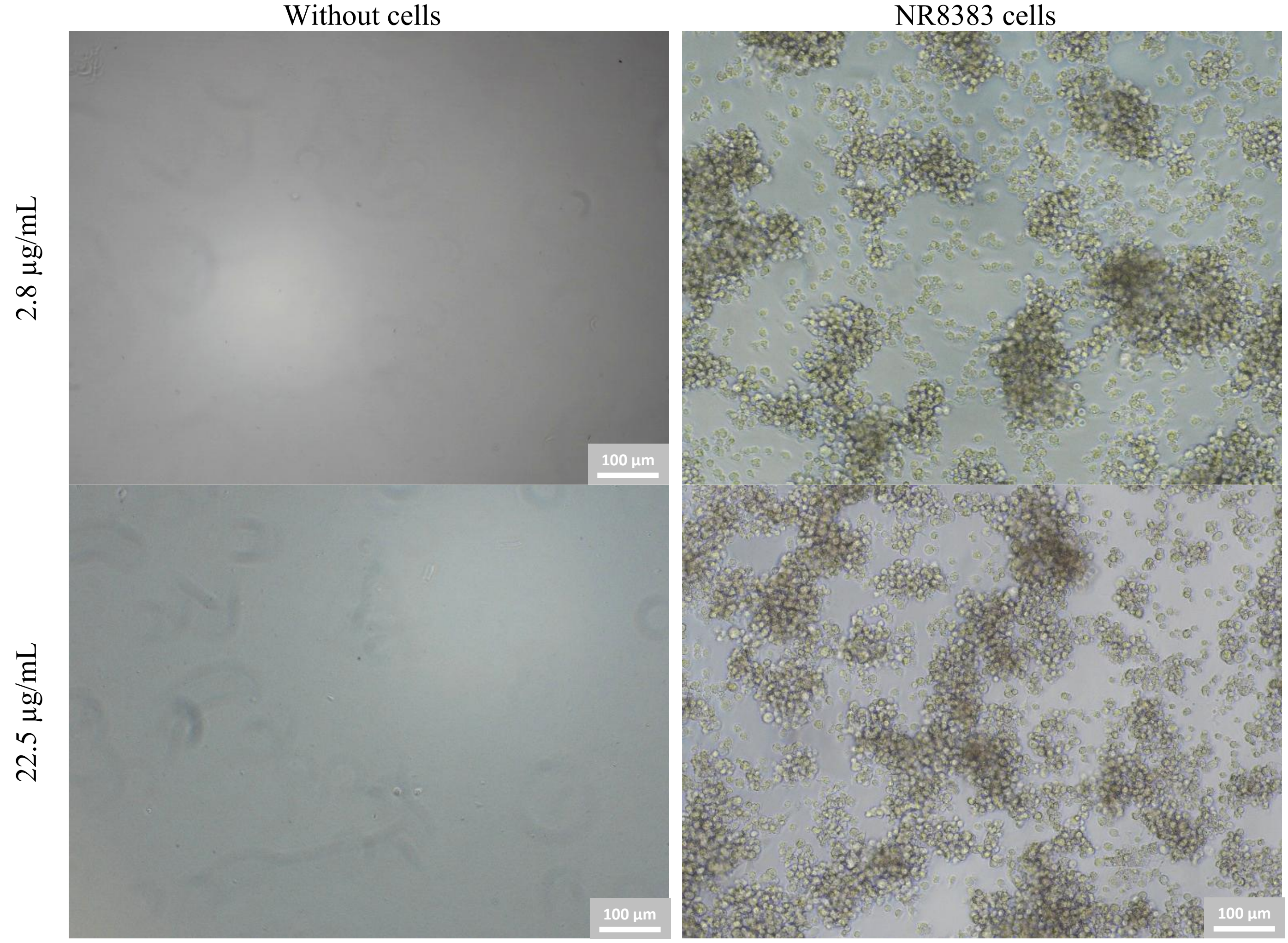

SiO_2 NM-203 were not visible in phase-contrast microscopy.

Lab1
Particle 8 - ZnO NM-111

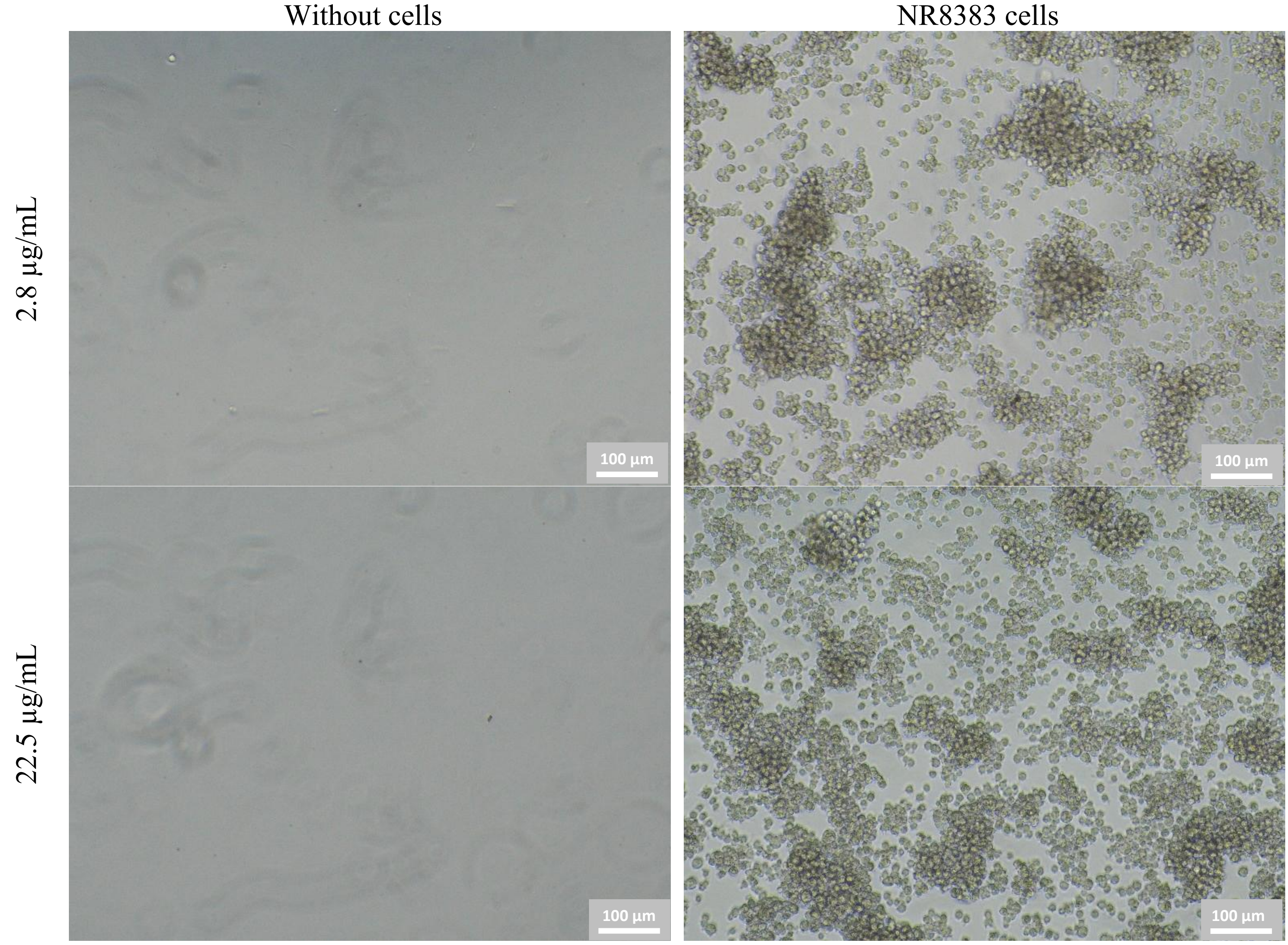

ZnO NM-111 were not visible in phase-contrast microscopy.

As at the Inhaled Particle

Lab1
Particle 9 - Quartz DQ12

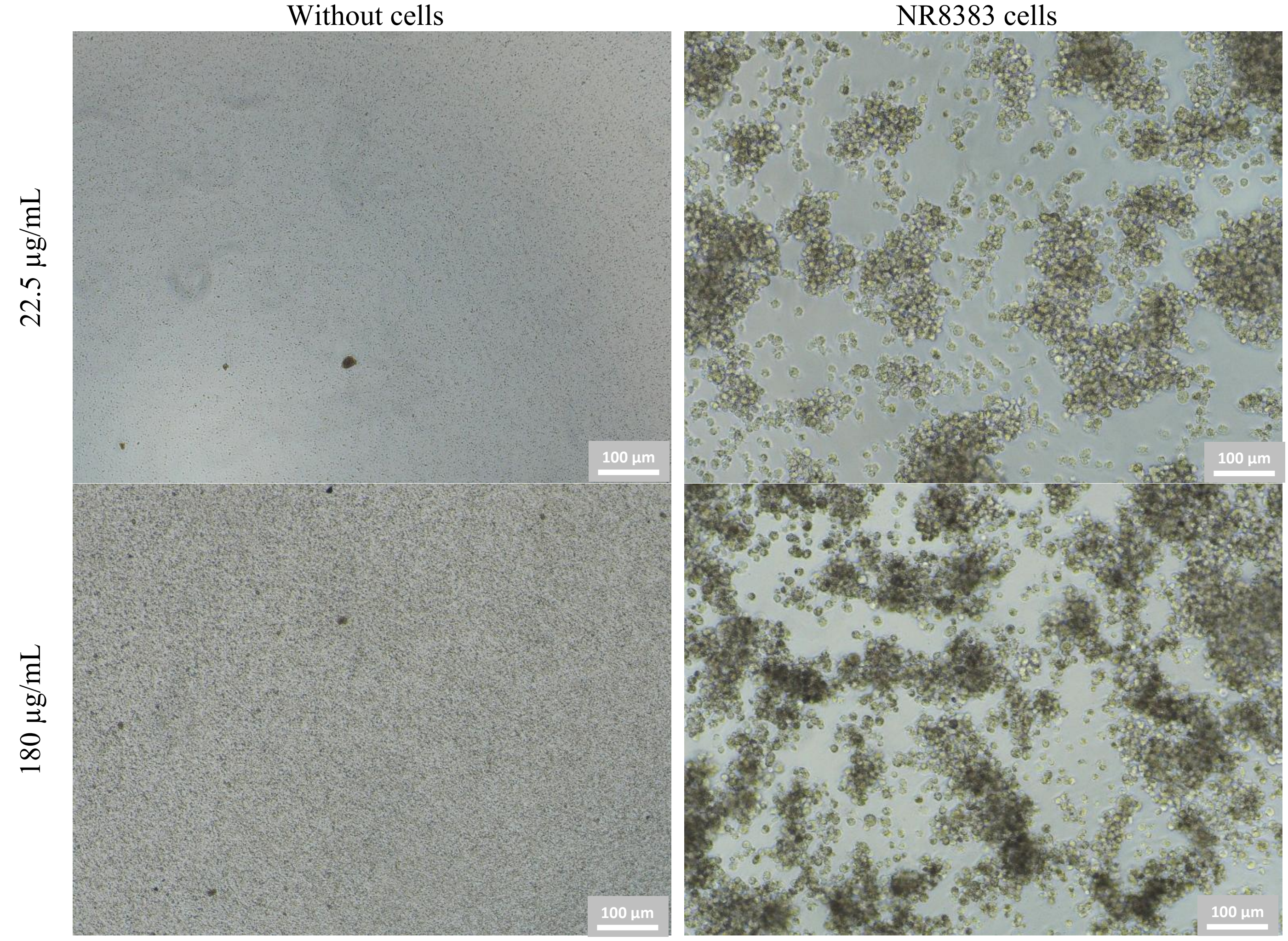

Lab1

Particle 10 - SiC Nanowires

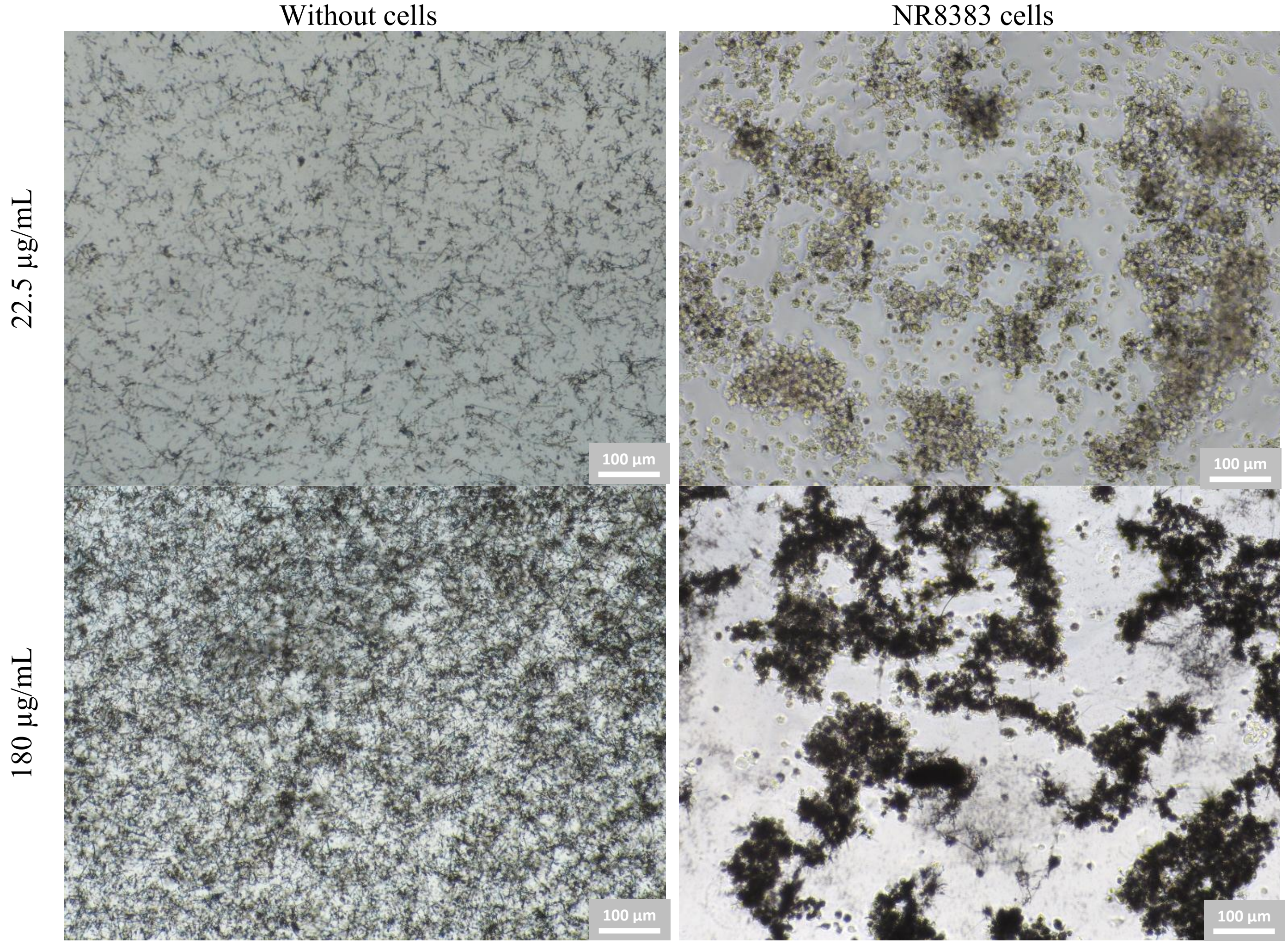

Lab1
Particle 11 - $BaSO_4$ NM-220

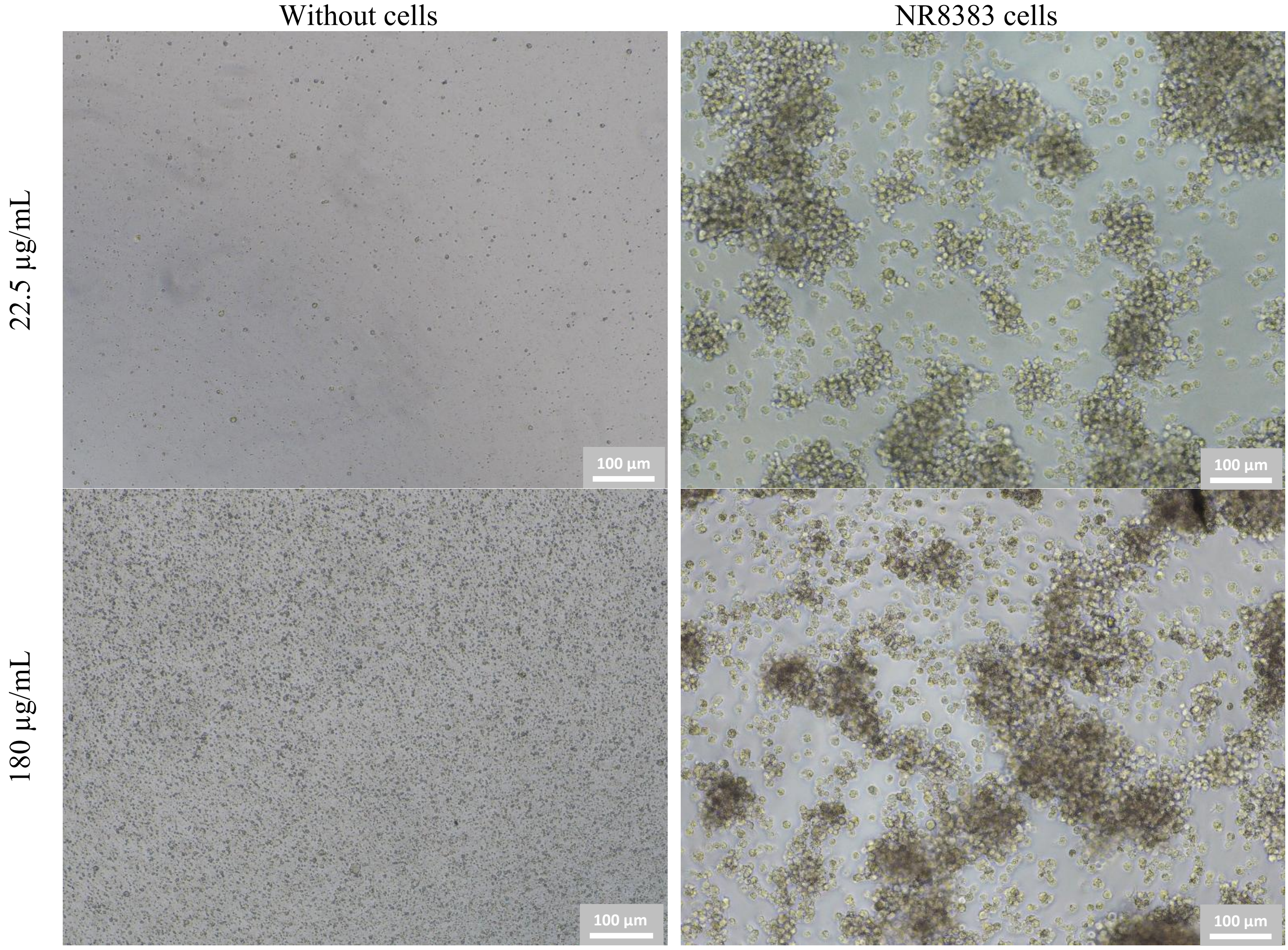

Lab1
Particle 12 - Corundum

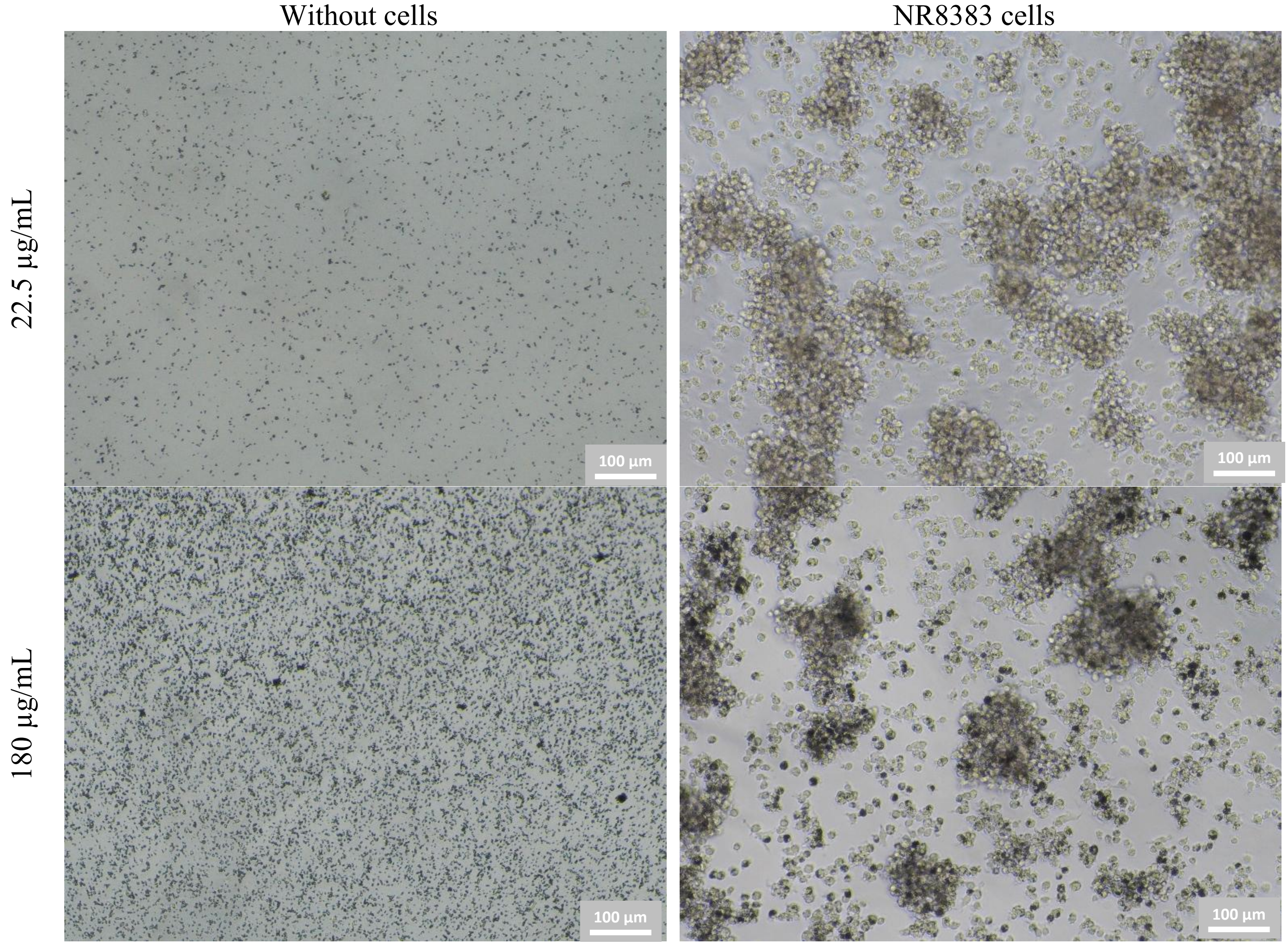

Supplement: Supplementary file 1 [file DataSheet1.pdf]
